# Rapid increase in West Siberia’s retrogressive thaw slumps since 1964 associated with Arctic winter warming

**DOI:** 10.1038/s41598-026-56146-9

**Published:** 2026-06-11

**Authors:** Nina Nesterova, Marina Leibman, Carl Stadie, Tobias Hölzer, Ingmar Nitze, Ilia Tarasevich, Kathrin Maier, Maiia Vasileva, Hugues Lantuit, Jonas Küpper, Guido Grosse

**Affiliations:** 1 Permafrost Research Section, Alfred Wegener Institute Helmholtz Centre for Polar and Marine Research, 14473 Potsdam, Germany; 2 Institute of Geosciences, University of Potsdam, 14469 Potsdam, Germany; 3 Earth Cryosphere Institute, Tyumen Scientific Centre SB RAS, Tyumen, 625026 Russia; 4 Faculty of Electrical Engineering and Computer Science, Technical University Berlin, 10623 Berlin, Germany; 5 Department of Earth and Atmospheric Sciences, University of Houston, Houston, TX 77004 USA; 6 Department of Environmental Engineering, ETH Zurich, 8093 Zurich, Switzerland

**Keywords:** Climate sciences, Environmental sciences, Natural hazards

## Abstract

Retrogressive thaw slumps (RTSs) are key indicators of permafrost thaw in the West Siberian Arctic. Based on a new high-resolution remote sensing-derived, field-verified inventory of 6168 RTSs for the Yamal and Gydan peninsulas, we provide the first large-scale spatio-temporal and climate sensitivity analysis from 1964 until 2024. In Gydan, RTS clusters are located at higher elevations, on rougher terrain, and in lake-rich areas, whereas such associations are weaker in Yamal. Temporal analysis of RTS using historical and modern satellite imagery for key sites (~ 6,103 km^2^) indicates that RTS numbers increased 23-fold from 1964 to 2024, and initiation rates rose 26-fold. A discrete-time Bayesian hazards model identified summer maximum precipitation as the strongest short-term factor. However, winter warming was the dominant long-term driver, with an increase of + 4.82 K resulting in a 29-pp increase in annual initiation probability. RTS development in the region poses high risks to gas and transport infrastructure.

**Supplementary Information:**

The online version contains supplementary material available at 10.1038/s41598-026-56146-9.

## Introduction

In recent years, evidence is mounting that Arctic winter warming is profoundly changing the terrestrial Arctic, including permafrost warming^1^, widespread talik formation^2^, and accumulating ecological impacts of episodic but strong winter warming events^3,4^. Permafrost, defined as ground that remains below 0 °C for at least two consecutive years^5,6^, underlies about 15% of the Northern Hemisphere landmass^7^. Arctic permafrost experiences thawing and a reduction in extent in response to climate warming^1,8^. Permafrost not only affects high northern latitude landscapes and ecosystems but also stores large amounts of carbon and nutrients, with carbon loss upon thaw contributing to global climate feedback^9^. What drives permafrost thaw and over which time scales is therefore important to understand.

**Fig. 1:**
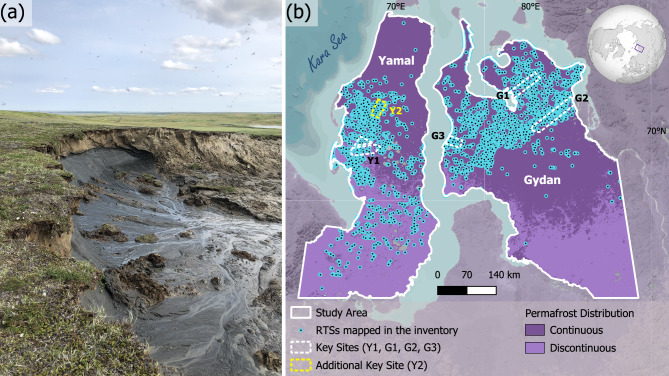
Retrogressive thaw slumps in West Siberian Arctic. **(a)** RTS in Central Yamal (photo by Nina Nesterova, July 2019). (**b**) Study area in the West Siberian Arctic: Yamal and Gydan peninsulas; RTSs mapped in the inventory by^16^,Dashed white and yellow polygons show the location of five key sites for time series analysis with high-resolution historical and recent imagery. Permafrost distribution is based on Obu et al.^17^. Basemap of panel (b): AWI Basemap v.0.9 (2020) ©2013–2025 Alfred-Wegener-Institut Helmholtz-Zentrum für Polar- und Meeresforschung. The map was created using QGIS Desktop v. 3.36.2 (see Methods).

Thaw of ice-rich permafrost often occurs abruptly, leading to the formation of distinctive landforms^10^. One of the most widespread and dynamic indicators of abrupt thaw is a retrogressive thaw slump (RTS)^11^. An RTS is a slope failure initiated by the exposure and thaw of massive ground ice^12^ (Fig. 1a). RTSs are reported throughout permafrost regions in the Arctic and high-mountains where they significantly alter topography, hydrology, soil, and vegetation, while mobilizing substantial quantities of sediments, carbon, and nutrients to downstream environments^13–15^.

These landforms evolve in a polycyclic fashion through phases of active ice ablation and mudflow, followed by stabilization, vegetation colonization, and further possible reactivation^12,18,19^. The initiation of RTSs was reported to be linked to climate drivers such as rising summer air temperatures and increased summer precipitation^20–22^. However, predicting areas of RTS initiation remains complicated due to the uncertainty of heterogeneous ground ice distribution^23,24^^,^ limited field data^25^^,^ and the lack of suitable models^26^.

The West Siberian Arctic is largely an accumulation plain composed of sandy and clayey Quaternary sediments and shaped into a dissected lowland with long, gentle slopes and dense ravine–gully and small-river networks formed by thermoerosion and thermokarst^27,28^. Permafrost in this Arctic region is mostly continuous^17^ (Fig. 1b). Massive ground ice is abundant and can be up to several tens of meters thick^29^. The Quaternary history in this region and the origin of massive ground ice are still debatable^30^. Nevertheless, the widespread near-surface occurrence of massive ground ice^31,32^ resulted in a noteworthy and impressive abundance of RTSs in this region^19,33^. The regional warming and deepening of the active layer catalyze further RTS initiation and expansion^8^^[,34,]^^35^.

Yet, this trend has not been documented at the western Arctic scale by field-verified studies. Most studies either focused on local field sites^19,33,34,36^ or aimed at automated large-scale mapping with clear limitations and uncertainties due to a lack of field verification^37–41^.

A recent high-resolution field-verified, manually-mapped, and classified RTS inventory dataset for the West Siberian Arctic now enables a large-scale robust analysis without compromising accuracy^16^. Here we present the first large-scale analysis of spatial and temporal patterns of RTS distribution in the West Siberian Arctic, particularly for the Yamal and Gydan peninsulas (Fig. 1), and an analysis of factors and drivers contributing to their accelerating development.

Our study aims to answer the following research questions: (1) What geomorphological and environmental conditions are associated with the spatial distribution and clustering of RTSs across the West Siberian Arctic? (2) How have RTS number, initiation rates, and headwall retreat rates changed from the 1960s to the present, and do these changes indicate accelerating permafrost thaw? (3) To what extent are long-term RTS development trends driven by climate forcing? and (4) How does ongoing RTS development interact with existing infrastructure and landscape systems, and what risks or vulnerabilities does this pose?

To answer the first research question, we analyzed RTS spatial clustering and its relation to elevation, water bodies, and landcover. To study temporal RTS dynamics since the 1960s, we analyzed historical as well as modern high-resolution satellite imagery for five key sites (Fig. 1). To estimate climate influence on RTS dynamics, we compared RTS temporal trends with different climate variables by implementing a Bayesian statistical modeling approach. To assess the risks of RTS development, we analyzed the current and possibly impacted infrastructure.

## Results and discussion

### Spatial distribution

To explore the spatial distribution of RTS clusters in the West Siberian Arctic, we aggregated RTS point locations from the inventory^16^ into hexagonal grid cells (Uber H3 grid, resolution 6, ~ 35 km^2^)^42^. All further calculations and statistics were aggregated per hexagonal grid cell.

**Fig. 2:**
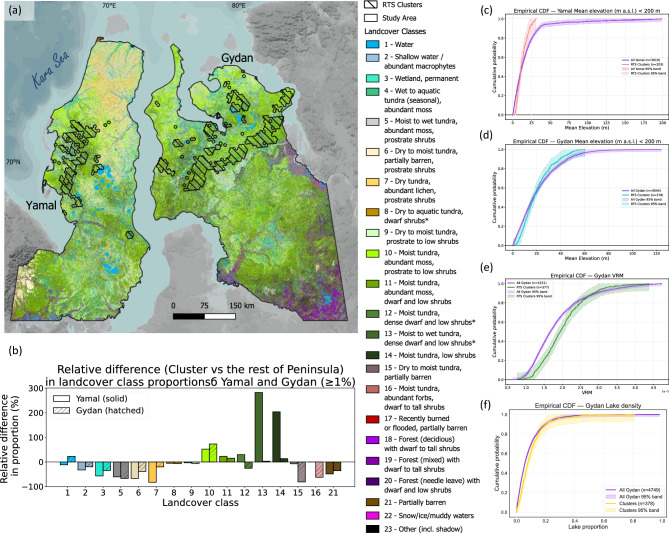
Spatial distribution of RTSs in West Siberian Arctic. **(a)** Landcover composition^44^ for the study area of both peninsulas and statistically significant RTS clusters (black shaded). (**b)** Relative difference in landcover class proportion for RTS clusters versus the rest of Yamal and Gydan peninsulas. (**c)** Empirical CDF of mean elevations (m a.s.l.) excluding Ural Mountains (> 200 m a.s.l.) for RTS clusters and the rest of the Yamal Peninsula. (**d)** Empirical CDF of mean elevations (m a.s.l.) excluding Ural Mountains (< 200 m a.s.l.) for RTS clusters and the rest of Gydan Peninsula. (**e)** Empirical CDF of VRM values for RTS clusters and the rest of the Gydan Peninsula. (**f)** Empirical CDF of lake density values for RTS clusters and the rest of the Gydan Peninsula. Basemap of panel (**a**): AWI Basemap v.0.9 (2020) ©2013–2025 Alfred-Wegener-Institut Helmholtz-Zentrum für Polar- und Meeresforschung. The map was created using QGIS Desktop v. 3.36.2 (see Methods).

Our results confirm previously reported spatial clustering of RTSs in the region^16^, with multiple statistically significant clusters revealed using Anselin Local Moran’s I (p ⋜ 0.05) (black hatched areas in Fig. 2a). These clusters represent 21,179 km^2^, accounting for 6.9% of the total study area for both peninsulas (7,160 km^2^—5.4% for Yamal,14,019 km^2^—8% for Gydan). This is consistent with a general tendency of spatial clustering of RTSs across multiple studies in the Arctic^39,43^.

The analysis of environmental characteristics of RTS clusters revealed differences between the Yamal and Gydan peninsulas.

Landcover analysis of RTS clusters on both peninsulas showed a dominant association with *moist tundra*, with only minor differences between Yamal and Gydan (Fig. 2a, b). In Yamal, RTS clusters occur mainly in *moist tundra with abundant moss* and *prostrate to low shrubs/dwarf and low shrubs* (classes 10, 11, 12; IDs here and below follow^44^, *moist to wet tundra with dense dwarf and low shrubs* (13), and *moist tundra with low shrubs* (14), together accounting for ~ 60% of the landcover hosting RTS clusters. In contrast, *dry tundra with abundant lichen and prostrate shrubs* (7), which dominates northern Yamal, is only scarcely associated with RTS clusters (Fig. 2a, b).

In Gydan, RTS clusters are likewise primarily associated with *moist tundra* classes, particularly *moist tundra with abundant moss and prostrate to low shrubs* (10), *moist tundra with low shrubs* (14), *dwarf and low shrubs* (11), and *moist to wet tundra with dense dwarf and low shrubs* (13). *Water* (1) is also among the dominant landcover classes for RTS clusters in Gydan. Combined, these classes make up ~ 70% of the landcover in Gydan RTS clusters. The most underrepresented class relative to its peninsula-wide extent is *moist tundra with abundant forbs and dwarf to tall shrubs* (16).

Elevation analysis revealed that in both peninsulas, mean elevation (less than 200 m a.s.l to exclude the Ural Mountains) for RTS clusters did not differ from non RTS clusters (U-test non-significant), but mean elevation distributions were significantly different according to Kolmogorov–Smirnov test (Yamal: D = 0.164, p < 0.001 after Bonferroni correction; Gydan: D = 0.108, p < 0.001 after Bonferroni correction). Most RTS cluster mean elevations (2.5th–97.5th percentiles) were between 3.4 m a.s.l. and 28.2 m. a.s.l. in Yamal, and between 3.8 m. a.s.l. and 50.0 m. a.s.l in Gydan.

For terrain roughness, expressed as the Vector Ruggedness Measure (VRM), RTS clusters in Yamal do not differ significantly from the rest of the peninsula. In Gydan, however, VRM values for RTS clusters are significantly higher (U = 874,296, p < 0.001 after Bonferroni correction; D = 0.218, p < 0.001 after Bonferroni correction), indicating rougher terrain (Fig. 2e). Most RTS cluster VRM values (2.5th–97.5th percentiles) were between 1.1e − 5 and 3.4e − 5, still representing generally smooth topography.

Lake density shows a similar pattern: no significant difference between RTS clusters and the rest of Yamal, but a strong, significant contrast was found in Gydan (U = 1,036,752, p < 0.001; D = 0.144, p < 0.001 after Bonferroni correction). In Gydan, RTS clusters are associated with substantially higher lake densities, as illustrated by the empirical CDF (Fig. 2f) and consistent with the overrepresentation of the water class (Fig. 2b). Most RTS cluster lake density values ranged from 0.002 to 0.373.

Our findings show that RTS clusters tend to occur on higher elevations and rougher terrain. This is generally consistent with previous findings of inland RTSs mostly occurring at slopes and transitions between landforms: river banks, lake shores, slopes of streams, and valleys^11^. Most of the RTS clusters in Gydan are located in the hilly ridge of Gydanskaya Gryada. The flat terrain found in the north of Yamal is characterized by the quasi- absence of RTSs (Fig. 1). This might be due to the prevalence of very low elevations (< 20 m a.s.l., see SI 1 -2), low VRM (1.65e-5, see SI 1—2), and the dominance of the Dry Tundra landcover class. The latter is characterized by lower soil moisture and higher mineral component as well as less pronounced subsidence, suggesting limited ground ice melt and lower soil moisture dynamics^44^.

The following parameters did not show any influence on RTS spatial distribution: 1) lake parameters such as area, perimeter, eccentricity, orientation, solidity; 2) the aspect of RTS in regard to the nearest lake; 3) the aspect of RTS in regard to the nearest river; 4) Quaternary geology composition based on the State Geological Map of the Russian Federation; 5) ground ice characteristics based on historical Soviet permafrost maps. Although lakes can indicate regions that once contained massive ground ice, their current morphology is no longer directly related to modern climatic or topographic conditions that control RTS formation. Since RTS development fundamentally depends on the presence of massive ground ice, local slopes, and climate, other parameters influence RTS distribution only if they determine or are determined by these key factors. The lack of relation between RTS spatial distribution and aspect is consistent with previous studies reporting contrasting or absent relationships between aspect and RTS activity in different permafrost regions (e.g.^37,45–47^). Another difficulty is the lack of a universal method for measuring aspect: some studies use the dominant slope aspect within the RTS, others the surrounding slope, and others the mudflow or its orientation towards nearby waterbodies. Finally, the absence of detectable influence from Quaternary geology and ground ice maps likely reflects the very coarse resolution of these historical datasets. The datasets, their limitations, and the conducted statistical tests are provided in SI 1–1.

### RTS dynamics and association with climate variables

**Fig. 3:**
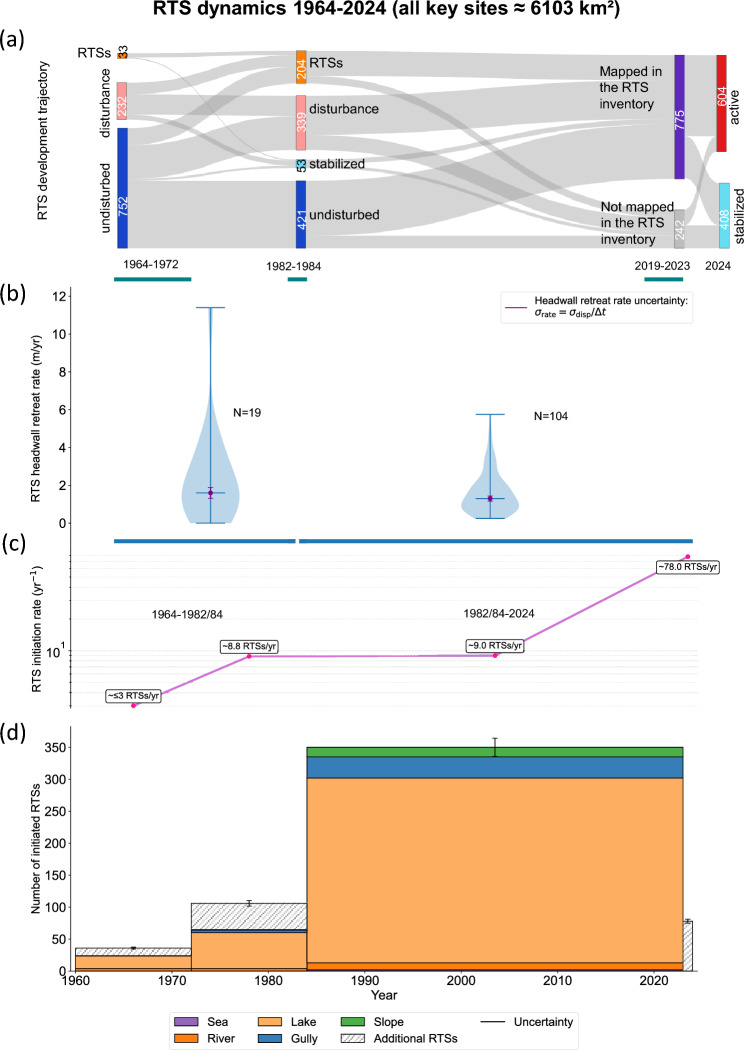
RTS dynamics at key sites in the West Siberian Arctic 1964–2024 (same x-axis of time for all plots a to d). **(a)** RTS development trajectory including categories of undisturbed tundra, disturbance feature, RTS, as well as stabilized disturbance for the periods 1964–1972 and 1982–1984. The period 2019–2023 represents the majority of the years of satellite images used in the ESRI basemap mosaic in the inventory by Nesterova et al.^16,48,49^. For 2024 only active or stabilized categories were distinguished. (**b)** Headwall retreat rate distribution over the periods of 1964–1982/84 and 1982/84–2024. (**c)** RTS initiation rates per year for the time periods of 1960–1972 (or earlier), 1972–1984, 1985–2023, and 2024. The total headwall retreat rates inherit the positional uncertainty of historical imagery georeferencing of ~ 4 m, which was recalculated for the displacement uncertainty over the years. (**d)** Number of initiated RTSs for the time periods of 1960–1972 (or earlier), 1972–1984, 1985–2023, and 2024. See Fig. 1 for location of key sites.

**Fig. 4:**
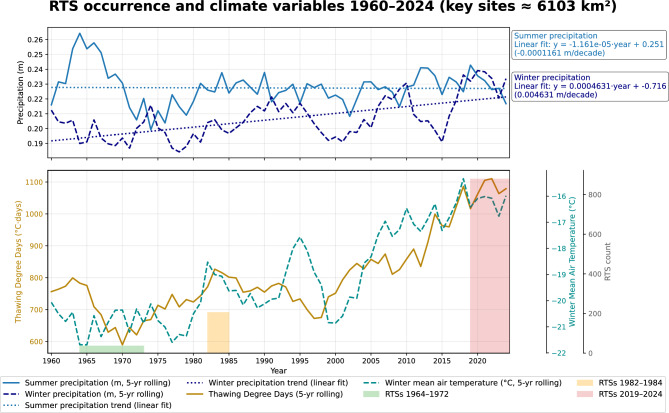
RTS occurrence and climate variables at key sites for 1964–2024 (all plots share the same x-axis): Mapped RTS count, summer (May–September) mean air temperature (°**C**), winter (October–April) mean air temperature(°**C**), total summer precipitation (**m**), total winter precipitation (**m**). See Fig. 1 for location of key sites.

With our analysis of RTS development for the 1964 to 2024 period, we found an overall 23-fold increase in the number of RTS for all key sites (~ 6103 km^2^) from the time period of 1964–1972 to 2024 (Figs. 3 and 4). Most of the increase in the number of RTS occurred after 1984, contributing 86% to the total increase (Fig. 3).

Initiation rates of RTS accelerated over time, with the first large spike taking place as early as the 1980s and the largest increase in the last thirty years reaching up to ~ 78 RTSs yr^−1^ (26-fold increase). Our findings of the RTS initiation rates of ⋜3 RTSs yr^−1^ in the 1960s and ~ 8.8 RTSs yr^−1^ for 1972–1982 suggest that the initiation rates were comparable with the ones reported for Canada. The reported RTS initiation rate at the Aklavik Plateau (360 km^2^, a > 16 times smaller study area) was 0.35 RTS yr^−1^ for the 1954–1971 time period and 0.68 RTSs yr^−1^ for 1985–2004^50^. Meanwhile, modern rates of RTS initiation of ~ 78 RTSs yr^−1^ for our area are comparable with those reported for current RTS hotspots. This includes, for instance, the neighboring Taymyr Peninsula, where one study in North Taymyr (68,000 km^2^, a > 10 times larger study area) reported ~ 120 RTS yr^−1^ for 2010–2021^51^. Another study focused only on the extremely active area of East Taymyr (91 km^2^) and identified rates of ~ 23 RTS yr^−1^ for the 2011–2020 period^52^. Comparable high initiation rates were reported for Banks Island (~ 70,000 km^2^, a > 10 times larger study area) in Canada from 1984 to 2013, with values of 136 RTS yr^−1^^53^.

We found that headwall retreat rates did not change much over time. The median headwall retreat rates were 1.3 m yr^−1^ for 1964–1982/84 and 1.6 m yr^−1^ for 1982/84–2024, and the highest headwall retreat rate for the most active single RTS was 11.4 m yr^−1^ during the 1964–1982/1984 time period (Fig. 3). These headwall retreat rates are similar to those reported for Tibetan RTSs (0.05–5 m yr^−1^)^54^. West Siberian RTS headwall retreat rates are rather stable and slow, especially if compared to the 6.2 m yr^−1^ estimated in the Canadian High Arctic^25^ or in the neighboring Yugorsky peninsula with 66 m yr^-1^^22^. The maximum headwall retreats were 256 m (1972–2024) in Y2 (SI 1—4) and 230 m (1984–2024) at key site G1 (SI 1—4). The relatively stable headwall retreat rates, despite the marked increase in RTS initiation, can be explained by several interacting factors. Retreat appears to be limited by local morphological and thermal constraints: once the initial rapid headwall retreat exposes zones with lower ice content and gentler slopes, the thaw potential becomes increasingly constrained. Sediment accumulation on the headwall and at its base can further insulate the ice, reducing energy transfer and slowing retreat unless removed by intense summer precipitation. In some cases, insulating covers or snowdrifts can delay active thaw. Thus, even as warming conditions have increased overall thaw potential, the actual retreat rate may have reached a threshold governed by slope, ice content, and sediment dynamics.

The analysis of the RTS development trajectory (Fig. 3a) aimed to define whether RTSs found in recent imagery are located in previously disturbed areas in the 1960s and 1980s. About 42% of all RTSs were found in areas of undisturbed tundra both in 1962–1972 and 1982–1984 time periods, while 33% were found as disturbance features in the 1980s, and 23% were disturbance features in the 1960s (Fig. 3). We show that a substantial part of RTSs occurred in pre-disturbed conditions back in the 1960s, which is similar to findings from the Canadian Arctic (Aklavik Plateau and Yukon Coast), where RTS locations were already observed in the 1950s^50,55^.

Figure 4 shows the increase in the number of mapped RTSs for the time periods of 1964–1972, 1982–1984, and 2019–2024. The climate data suggests an increase in both winter (October–April) and summer (May–September) air temperatures, with the winter air temperature warming at a much faster rate (~ 0.674 °C/decade) than summer (~ 0.285 °C/decade). There is also a small yet significant increase in winter precipitation (0.004 m/decade; Fig. 4).

To study the sensitivity of RTS initiation to climate variables, we employed a discrete-time proportional-hazards model^56^. The model assesses the probability of RTS initiation, assumed to come only from climate covariates. The results for the first time period (1964–1984) and the second time period (2019–2024) suggest that summer maximum precipitation and winter mean air temperature are the most important short-term factors for RTS initiation. An increase in summer maximum precipitation by one standard deviation raised the annual RTS initiation probability by 29.76 percentage points (pp) (95% Bayesian uncertainty interval 28.64–30.78) with a hazard ratio (HR) of 4.41 (3.89–4.90), making it the strongest short-term influencing factor. Summer rainfall is reported to be associated with thaw-related mass movements not only in West Siberian Arctic^57^ but also in the Canadian Arctic^20,53^ and Alaska^58^.

The increase in one standard deviation of winter mean air temperature raises the probability of annual RTS initiation by 18.25 pp (16.06–20.46) with an HR of 2.97 (2.65–3.34), indicating strong preconditioning. Moreover, the winter mean air temperature over the two preceding years (“memory” climate variable that stores the cumulative information of the last two winters) has a rather small but still strongly evident effect on RTS initiation probability, adding 2.91 pp (0.89–4.93) with a one-standard-deviation increase. Other climate variables have rather small effects or evidence.

Our results suggest that winter mean air temperature is a dominant long-term driver of RTS initiation. The increase in winter mean air temperature by 1.62 standard deviations leads to a rise in annual RTS initiation probability over the long term by 29.65 pp. The other analysed climate variables were found to be of weak influence (Table 1).

**Table 1:** Covariate effects on annual initiation for two short time periods (1964–1984 and 2019–2024) as well as for one long time period (1964–2024). Analysis conducted for a + 1 standard deviation increase (risk-set AMEs* in pp, with 95% HDIs*; HRs* as medians with 95% intervals). Bayes factors are Savage–Dickey BF_10_*.

1964–1984 and 2019–2024 time periods					
Covariate	Δγ* (pp)		HR (median [95%])		BF_10_
Winter mean air temperature	+ 18.25 [16.06, 20.46]		2.97 [2.65, 3.34]		≫ 10^12^
Summer maximum precipitation	+ 29.76 [28.64, 30.78]		4.41 [3.89, 4.90]		≫ 10^12^
Winter maximum precipitation	+ 0.98 [0.12, 2.10]		1.04 [1.00, 1.11]		0.12
Summer mean air temperature	+ 1.79 [-0.22, 3.52]		1.14 [1.02, 1.28]		0.57
Memory: winter maximum precipitation (2 years)	+ 0.86 [0.00, 2.23]		1.05 [1.00, 1.14]		0.32
Memory: winter mean air temperature (2 years)	+ 2.91 [0.89, 4.93]		1.23 [1.09, 1.37]		17.95
Long-term period 1964–1984 → 2019–2024					
Covariate		1964–1984 → 2019–2024 change (natural; SD*)		Implied Δγ (pp, linear)	
Winter mean air temperature		+ 4.82 °C (+ 1.62 SD)		+ 29.65	
Summer maximum precipitation		0.01 m (+ 0.26 SD)		+ 7.87	
Winter maximum precipitation		+ 0.05 m (+ 1.43 SD)		+ 1.41	
Summer mean air temperature		+ 3.17 °C (+ 2.28 SD)		+ 4.1	

AME—Average marginal effect, an absolute change in the annual initiation probability; HDI—Highest density interval, a Bayesian uncertainty interval; HR—hazard ratio, the ratio of the hazard rates; Savage–Dickey BF_10_—Bayes factor (BF)—evidence estimate, BF > 10 indicates strong evidence; SD—standard deviation; pp—percentage points, an absolute change on the probability scale; Δγ—the change in RTS initiation probability.

Multiple studies reported warm summer air temperature being associated with RTS development^25,50,53,58,59^. Our findings suggest that the mean winter air temperature is not only a significant factor influencing RTS initiation over short time periods, but also a long-term driver. Winter air temperatures in the West Siberian Arctic increased much faster than in summer (Fig. 4), which is consistent with global Arctic winter warming that exceeds summer warming by at least four times^60^. This pronounced winter warming, in turn, implies a later freeze of the active layer, higher ground temperature and thus, thickening of the active layer, which was reported to deepen in the West Siberian Arctic over the last 50 years^35^. Warming winters also imply earlier snowmelt and thus, exposing the surface, which in its turn reduces the albedo earlier and leads to more heat transfer that warms the permafrost. Warming of permafrost as well as deepening of the active layer thickness were reported to activate thaw-related mass movements in Central Yamal^34^.

### Implications for current and future RTS development

**Fig. 5:**
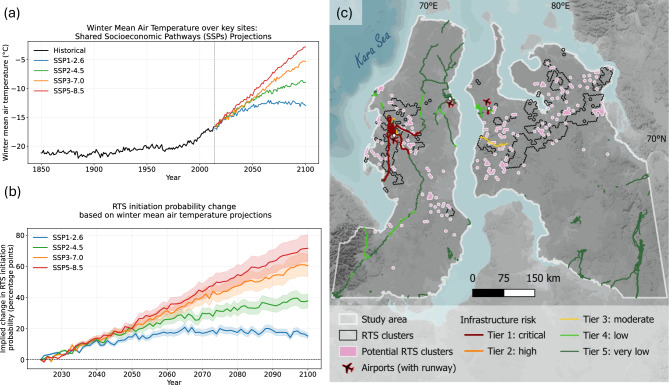
Implications of RTS development. **(a)** Projections on winter mean air temperature change by 2100 for key sites (see Fig. 1 for location of key sites), mean of CMIP6 ensemble subset (SI 2, 1.12). **(b)** Implied increase in RTS initiation probability according to winter mean air temperature raise. **(c)** Map of current and potential future RTS cluster areas and related tiered infrastructure risk (SI 2, 1.11). Basemap: AWI Basemap v.0.9 (2020) ©2013–2025 Alfred-Wegener-Institut Helmholtz-Zentrum für Polar- und Meeresforschung. The map was created using QGIS Desktop v. 3.36.2 (see Methods).

We anticipate more RTSs to occur in the West Siberian Arctic with ongoing climate warming and deepening of the active layer in the future. Projected warming of winter mean air temperature for the key sites ranges from 2.48 to 11.67 °C, depending on CMIP6-scenario (shared socioeconomic pathways) (Fig. 5a)^61,62^. Based on our findings, this winter air warming may imply an increase in RTS initiation probability between 15.23 and 71.59 percentage points (Fig. 5b).

To identify areas of possible future RTS formation in the region, we analyzed which areas share the same environmental parameters as current clusters (see Methods). We obtained potential future RTS cluster areas for Yamal totaling 3,127.3 km^2^, or 2.36% of the entire Yamal Peninsula (Fig. 5c), which would result in an increase of RTS cluster areas by 43.68%. The potential future RTS cluster areas for Gydan comprised 4,910.1 km^2^, or 2.28% of the entire Gydan Peninsula (Fig. 5c), representing an increase of RTS cluster areas by 35.02%.

Thaw-related mass wasting processes across the Arctic were reported to pose a risk to infrastructure stability^63,64^. To estimate the potential risk from RTS development to infrastructure, we analyzed the infrastructure currently impacted by RTS clusters, as well as where infrastructure overlaps with areas that may develop into RTS-clusters in the future. We classified infrastructure by criticality to the community and by overlap with current or potential RTS clusters or the vicinity (< 300 m) to current RTS. This classification resulted in five tiers: from critical (1) to very low risk (5) (see SI 2, 1.11).

The infrastructure in Yamal was found to be at much higher risk compared to Gydan: 99% of infrastructure classified as Tier 1 and 2 is located in Yamal. Infrastructure of Tier 1 in Yamal comprises ~ 1056 km of linear infrastructure and ~ 1.4 km^2^ of infrastructure area-wise. The largest type of affected infrastructure in Tier 1 is related to pipelines (Bovanenkovo-Ukhta pipeline) that comprise 718.7 km of main pipelines and related branches. Local roads and bridges within the Bovanenkovo gas field settlement classified as Tier 1 risk comprise 211 km. Around 73.5 km of the Obskaya-Bovanenkovo-Karskaya railway line were defined as Tier 1. Electrical lines as well as transmission substations in Tier 1 in the Bovanenkovo gas field settlement comprised 51.8 km. Tier 2 infrastructure in Yamal comprised 14.2 km of roads and 0.2 km^2^ of infrastructure area-wise, both also found in the Bovanenkovo gas field settlement. Around 309.1 km of local roads in Yamal were found to have only moderate risk (Tier 3).

Unlike in Yamal, the infrastructure of the Gydan peninsula is currently not impacted. There, Tier 1 infrastructure includes 11 km of local roads in the vicinity (< 300 m) of current RTSs. Tadebya-Yakha, a small settlement on the western coast of Gydan, as well as ~ 83 km of the local road to this settlement (classified as Tier 3) are located within the current and potential future RTS clusters. Gydan’s largest gas and oil field (Salmanovskoye), including the airport with a runway, is in the vicinity (~ 5 km) of potential future RTS clusters.

The infrastructure classified as Tier 1 and 2 (mostly related to gas extraction in Bovanenkovo) with heightened exposure to RTS clusters will pose substantial environmental risks for very vulnerable Arctic ecosystems. RTS development implies risk not only for the infrastructure but also for indigenous communities. Especially for those communities engaged in reindeer herding and seasonal migration, the transition from stable tundra surfaces to unstable, slumping terrain can complicate migration routes, increase travel hazards, reduce the fishery in the affected lakes, and affect the forage base for reindeer herding.

## Methods

### Spatial analysis

To perform all geospatial operations as well as map layout, we used Python language v.3.11.10 in Visual Studio Code v. 1.103.2 and QGIS v. 3.36.2. All geospatial operations and mapping were performed in WGS84 / UTM zone 43 projection (EPSG:32,643). To investigate the spatial distribution of RTSs as well as environmental parameters (elevation, lake density, landcover) within the study area, we aggregated our data into an Uber H3 hexagonal grid with a resolution of 6, which is ~ 35 km^2^^42^. This grid system was chosen since it is widely used^40,65^, well-integrated in Python, and fully reproducible due to its set boundaries.

To estimate elevation, we used the ArcticDEM dataset version 4.1 at 2 m resolution^66^. To assess lake density, we created a lake dataset based on the OpenStreetMap (OSM) dataset^67^. To analyze landcover, we used a new circumarctic land cover dataset developed specifically to better differentiate Arctic land cover types^44^. The infrastructure dataset was also accessed via OSM.

To study the dynamics of RTSs from 1964 to 2024, we analyzed historical high-resolution satellite imagery of CORONA (e.g.^68^ and HEXAGON (e.g.^69^ as well as modern PlanetScope satellite imagery (Planet Team, 2018). We mapped and classified not only RTSs from the existing inventory^16^, but also identified additional RTSs (53 RTSs on historical imagery and 78 new RTSs in 2024) that were missed. Dataset features were classified as “RTS” (visually clearly identified RTS with no doubts), “disturbance feature” (uncertain on the type of permafrost disturbance), “undisturbed tundra”, “stabilized disturbance” (in historical imagery visible as previously tundra disturbance or RTS, currently no visible disturbance), and, in PlanetScope 2024 imagery, as “active” or “stabilized”. RTS polygons for the 1960s and 1980s were digitized based on georeferenced CORONA and HEXAGON imagery^68,69^. RTSs were mapped and classified for years with available image acquisitions (1964, 1969, 1972, 1977, 1982, 1984) at four key sites: three in Gydan (G1, G2, G3) and one in Yamal (Y1) (Fig. 1). Key sites in Gydan covered ~ 4,489 km^2^ (~ 18% of RTS clusters in Gydan), and the key site in Yamal covered ~ 1,614 km^2^ (~ 10% of RTS clusters in Yamal). The key sites were selected based on the availability of multi-temporal historical imagery for the 1960s and 1970s. For an additional key site (Y2), historical imagery was only available for one time period. Hence, we include the results of its RTS dynamics only in the Extended Results in SI 1 (Sect.  4). RTS mapping and classification approaches are described in detail in Extended Methods in SI 2 (Sects. 1.6–1.8). We used manual mapping to resolve RTS occurrence and dynamics. Since estimating the uncertainty of manual mapping is not feasible, we applied the uncertainty range that was derived from an RTS mapping experiment performed in a previous work with the West Siberian RTS inventory^16^. The associated operator subjectivity of RTS misclassification was found to be 4.1%. RTS polygons for 2024 were created based on PlanetScope imagery^70^.

A detailed description of all datasets used in this study, the limitations, and all the pre-processing steps applied are described in detail in SI 2 (Sects. 1.1–1.4).

### Statistical analysis and filtering

Clusters of RTS accumulation were identified using Anselin Local Moran’s I statistic^71^, with Queen’s Case^72^ employed as the conceptualization of spatial relationships to account for adjacency effects. To select a methodology for hypothesis testing we first estimated the normality of analyzed distributions of mean elevations, VRM and lake density using multiple tests such as Shapiro–Wilk test^73^, D’Agostino’s K^2^ test combines skewness and kurtosis^74^, and two modifications of Kolmogorov–Smirnov test: Anderson–Darling test that puts more weight to tails^75^^,^ Lilliefors test that accounts for the fact that the mean and variance are estimated from the sample^76^. All of the tests eventually showed that distributions of all parameters, both for all the peninsula and for clusters, were not normal,thus, for the hypothesis testing, we chose a non-parametric test. To get complementary evidence, we applied two non-parametric tests: the Mann–Whitney U-test, which compares central tendencies through differences in medians^77^, and the Kolmogorov–Smirnov test that compares overall distribution differences, including shape, spread, and medians of the data^78^.

To determine where RTS could develop, we identified areas whose environmental parameters match those observed in current clusters. First, we filtered only areas with at least one RTS already mapped in Nesterova et al.^16,48,49^ which indicates the presence of massive ground ice. Next, we applied filters of the 95^th^ percentile of the value range for the parameters that were found to be significantly different in the clusters compared to the rest of the peninsula (see Results—Spatial distribution). For the Yamal Peninsula, the filtering was performed by mean elevation and proportion of landcover classes, while for the Gydan Peninsula, in addition to these two factors, VRM and lake density were also included.

### Bayesian climate hazard modelling of retrogressive thaw slump initiation

We analyzed the climate sensitivity of retrogressive thaw slump (RTS) initiation using a discrete-time proportional hazards model^79^. The choice of this model is explained by the need to adequately treat the gaps in the RTS data. Despite the relatively low number of RTS initiations in the 1964–1984 period, the effective sample size in the discrete-time proportional hazards model is deemed adequate. In contrast to purely correlative approaches that only relate yearly observed RTS counts to climate, a Bayesian hazards framework uses all sites covered by the datasets, including the many locations and years where no RTS was observed. The effective sample size is thus defined by the total number of observed locations over time, which remains constant across the whole study period. Annual initiation hazards were linked to covariates through a complementary log–log function^79^. The linear predictor included four standardized climate covariates: thawing degree days, summer maximum precipitation, winter mean temperature, and winter maximum precipitation. Coefficients expected to increase risk were constrained to be non-negative with HalfNormal priors. Models were estimated in a Bayesian framework with Hamiltonian Monte Carlo^80^. Prior predictive simulations confirmed plausibility, and posterior predictive checks compared yearly onset counts with observations (Auger-Méthé et al., 2019). Effect sizes are reported as hazard ratios (HR) and as average marginal effects (AME), expressed as percentage-point changes in annual initiation probability averaged across the pre-initiation risk set. For more detailed settings, see SI 2 (Sect. 2).

## Supplementary Information

Supplementary Information 1.

Supplementary Information 2.

## Data Availability

Georeferenced historical CORONA and HEXAGON images for key sites in the West Siberian Arctic are available at the PANGAEA data archive^49^, 10.1594/PANGAEA.987396). The dataset of classified and delineated retrogressive thaw slumps in five key sites in the West Siberian Arctic in the 1960s, 1970s, 1980s, and 2024 is also available at PANGAEA^48^, 10.1594/PANGAEA.987395).

## Code availability

The codes that were created during the study are available on GitHub under https://github.com/carlstadie/Nesterova_et_al.
