# Supplementary material for: Rapid increase in West Siberia’s retrogressive thaw slumps since 1964 associated with Arctic winter warming
Source: Sci Rep. 2026 Jun 11;16:18164. doi: 10.1038/s41598-026-56146-9 (PMC13254099; doi:10.1038/s41598-026-56146-9)
Supplement: Supplementary file 1 — Supplementary Information 1. [file 41598_2026_56146_MOESM1_ESM.docx]

**Supplementary Information 1**

**Extended Results**

**for**

**Rapid increase in West Siberia’s retrogressive thaw slumps since 1964 associated with Arctic winter warming**

Nina Nesterova^1,2^, Marina Leibman^3^, Carl Stadie^1,4^, Tobias Hölzer^1,2^, Ingmar Nitze^1^, Ilia Tarasevich^5^, Kathrin Maier^6^, Maiia Vasileva^1,2^, Hugues Lantuit^1,2^, Guido Grosse^1,2^

1. Permafrost Research Section, Alfred Wegener Institute for Polar and Marine Research, 14473 Potsdam, Germany
2. Institute of Geosciences, University of Potsdam, 14469 Potsdam, Germany
3. Earth Cryosphere Institute, Tyumen Scientific Centre SB RAS, 625026, Tyumen, Russia
4. Faculty of Electrical Engineering and Computer Science, Technical University Berlin, 10623 Berlin, Germany
5. Department of Earth and Atmospheric Sciences, University of Houston, TX 77004, United States
6. Department of Environmental Engineering, ETH Zurich, 8093 Zurich, Switzerland

Correspondence to: Nina Nesterova, [nina.nesterova@awi.de](mailto:nina.nesterova@awi.de)

**Contents of this file**

Figures S1-1 - S1-13

Tables S1-1 – S1-2

##

## 1. Results of parameters having no association with RTS distribution

Here, we present below the results from the analysis of parameters that showed no association with RTS distribution.

**Table S-1-1. Results of no association with RTS distribution**

| **Parameter** | **Data Source** | **Analysis** | **Result** |
| --- | --- | --- | --- |
| *Water Body Parameters* | | | |
| a) Lake Area | Lake dataset – described in SI 2 – Extended Methods - Waterbodies | To find whether the distribution of lake parameters of lakes with RTS and lake parameters of lakes without RTS differ, we used the non-parametric Kolmogorov-Smirnov (KS) test (Massey, 1951) | KS=0.557717,  Result: No difference (p ≥ 0.05) |
| b) Lake Perimeter |  |  | KS=0.528956,  Result: No difference (p ≥ 0.05) |
| c) Lake Eccentricity |  |  | KS=0.207343,  Result: No difference (p ≥ 0.05) |
| d) Lake Orientation |  |  | KS=0.079426,  Result: No difference (p ≥ 0.05) |
| e) Lake Solidity |  |  | KS=0.167083,  Result: No difference (p ≥ 0.05) |
| f) RTS Bearing towards the lake |  | We computed the bearings of RTS (as a point from the inventory) located at a lake shore to the nearest lake. To test whether the bearing is randomly distributed or has a certain pattern, we used the Rayleigh test (Mardia and Jupp, 2000) | Rayleigh test statistic: 0.0542  Result: The distribution is random (p ≥ 0.05) |
| g) RTS Bearing towards the river | River dataset – described in SI 2 – Extended Methods - Waterbodies | We computed the bearings of RTS (as a point from the inventory) located at a riverbank to the nearest river. To test whether the bearing is randomly distributed or has a certain pattern, we used the Rayleigh Test (Mardia and Jupp, 2000) | Rayleigh test statistic: 0.4892  Result: The distribution is random (p ≥ 0.05). |
| *Subsurface Parameters* | | | |
| a) Quaternary Geolog*y* | State Geological Map of the Russian Federation (new series), 2000a; 2000b, 2000c, 2004.  State Geological Map of the Russian Federation (third generation), 2014. | For visual comparison, we used georeferenced State Geological Maps of 1: 1 000 000 scale to extract the structure of Quaternary strata and the spatial distribution of the predominant Pliocene-Quaternary sediments in the north of West Siberia. We then georeferenced 5 sheets to the ArcGIS World Imagery basemap, fully digitizing the spatial distribution of sediments as a polygon dataset. | No relationship between sediment age and RTS distribution was revealed. |
| b) Ground Ice Content | Map of genetic types and ice content of the upper 10-meter section of permafrost in the West Siberian Plate, 1982 | For visual comparison, we used a map classifying frozen sediments of West Siberia into three types based on their ice content: low (I < 0.2), medium (0.2 < I < 0.4), and high (I > 0.4). We georeferenced this map to our regions based on the ArcGIS World Imagery basemap. | No spatial patterns were revealed between RTS distribution and the ice content due to constitutional ice. On the eastern coast of Yamal and the northern coast of the Gydan Peninsula, RTS clusters are located within the areas of high ice content due to ice wedges. |
| *c)* Cryolythology | Cryolythological Map of the USSR, 1985 | For visual comparison, we used a map classifying the territory of the USSR based on the predominant type of cryogenic structure. We georeferenced a part of the map corresponding to our region based on the ArcGIS World Imagery basemap. | No spatial patterns revealed |

## 2. Spatial distribution results

##
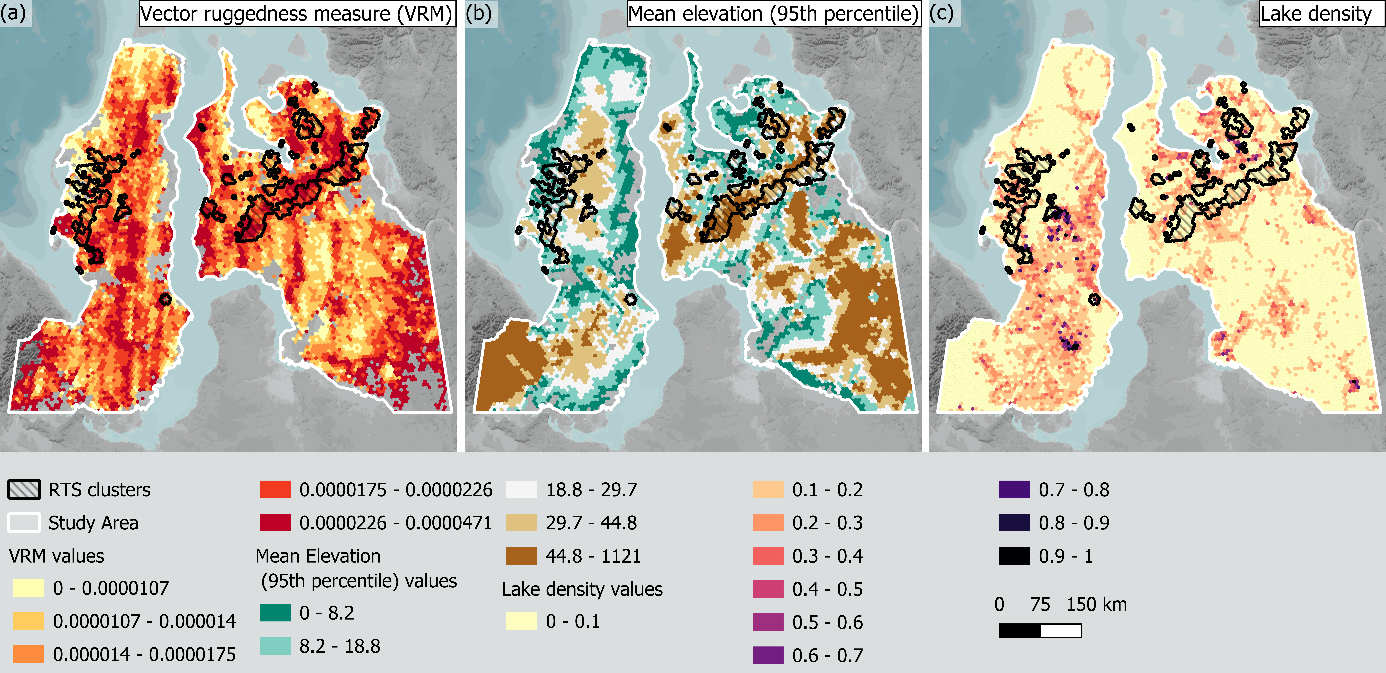


***Figure S-1-1: Spatial distribution of RTS clusters vs. environmental parameters: (a)*** *Vector ruggedness measure (VRM). (****b)*** *Mean elevation per H3 res=6 hexagonal grid cell (95^th^ percentile).* ***(c)*** *Lake Density. Basemap of (a): AWI Basemap ©2013-2025 Alfred-Wegener-Institut Helmholtz-Zentrum für Polar- und Meeresforschung. The map was created using QGIS Desktop v. 3.36.2 (see Methods).*

## 3. Description of the key sites

The key sites that were analyzed for RTS dynamics over 60 years have the following characteristics.

**Table S-1-2. Key site characteristics.**

| Key Site | Area (km^2^) | Area of key site covered by clusters (%) | Mean Lake density | Mean of Mean Elevation (95^th^ percentile) | Mean of VRM | Years analyzed and satellite |
| --- | --- | --- | --- | --- | --- | --- |
| G1 (Gydan) | 1420.514 | 4.4 | 0.17 | 40.97 | 6.55e-18 | 1964 (CORONA); 1984 (HEXAGON); 2024 (PlanetScope) |
| G2 (Gydan) | 2402.740 | 10.1 | 0.09 | 72.29 | 7.11e-18 | 1964 (CORONA); 1984 (HEXAGON); 2024 (PlanetScope) |
| G3 (Gydan) | 665.424 | 3.5 | 0.04 | 45.90 | 7.17e-18 | 1972 (HEXAGON); 1977(HEXAGON); 1984(HEXAGON); 2024 (PlanetScope) |
| Y1 (Yamal) | 1614.073 | 9.7 | 0.1 | 30.00 | 6.74e-18 | 1969 (CORONA); 1984 (HEXAGON); 2024 (PlanetScope) |
| Y2 (Yamal) | 820.264 | 8.3 | 0.05 | 41.76 | 5.30e-18 | 1972 (HEXAGON); 2024 (PlanetScope) |

## 4. RTS dynamics at key sites

The main text of the article includes the results on RTS dynamics aggregated over several time periods. Here, we present key site-specific results since they have some interesting regional variability (Figures S-1-2 - S-1-7). For example, there were almost no observed RTSs in Y1 and G2 key sites compared to others. The majority of maximum headwall retreats were found after 1984 (key sites G1, G2, Y1), while the maximum headwall retreats in G2 key sites were also found in the 1970s. RTS development trajectory suggests that the RTSs at the key sites were either mostly undisturbed (key sites G2, G3, Y1) back in the 1960s, or half-disturbed, like in the G1 key site. Yet, the Y2 key site showed a high number of permafrost disturbances and RTSs already in 1972. The comparison of area-balanced disturbance and RTS density in the historical imagery suggested that the Y2 key site was the most disturbed among other key sites (Figure S-1-7).

**
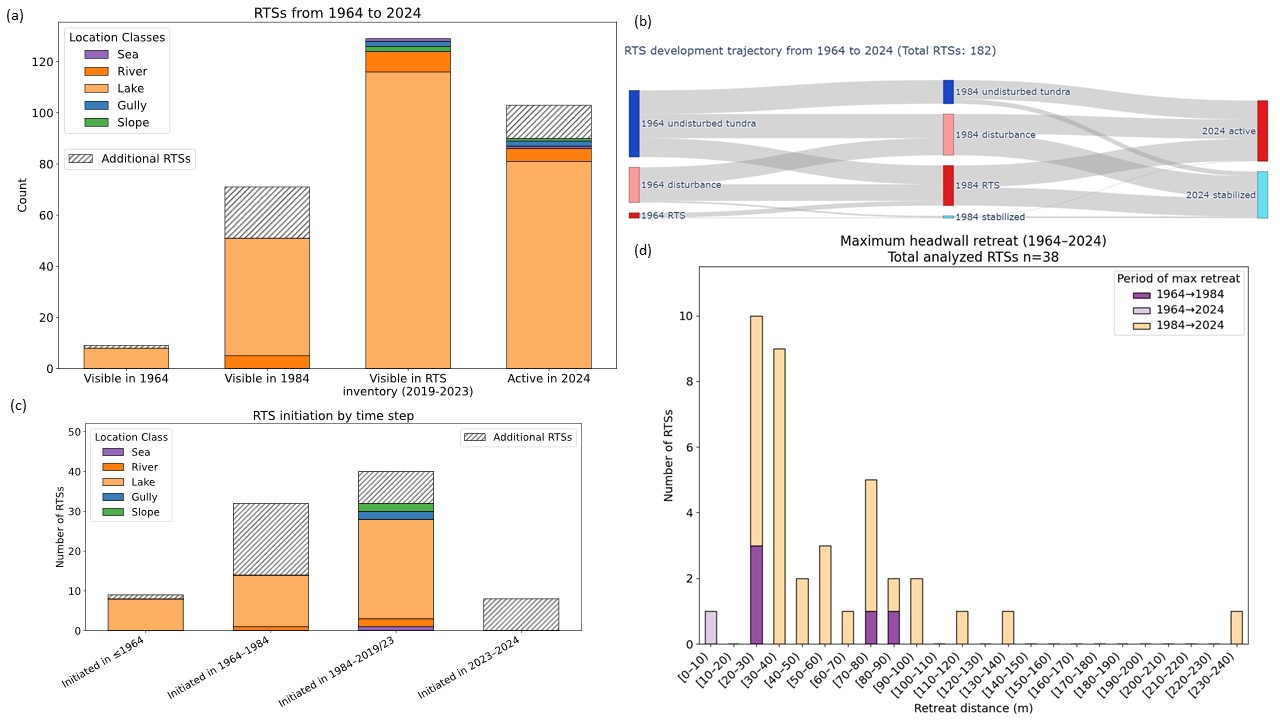
**

***Figure S-1-2: G1 key site RTS dynamics results: (a)*** *Counts of visible RTSs.* ***(b)*** *RTS development trajectory.* ***(c)*** *RTS initiation by time period.* ***(d)*** *Results of maximum headwall retreat color-coded by the time period of maximum retreat.*

**
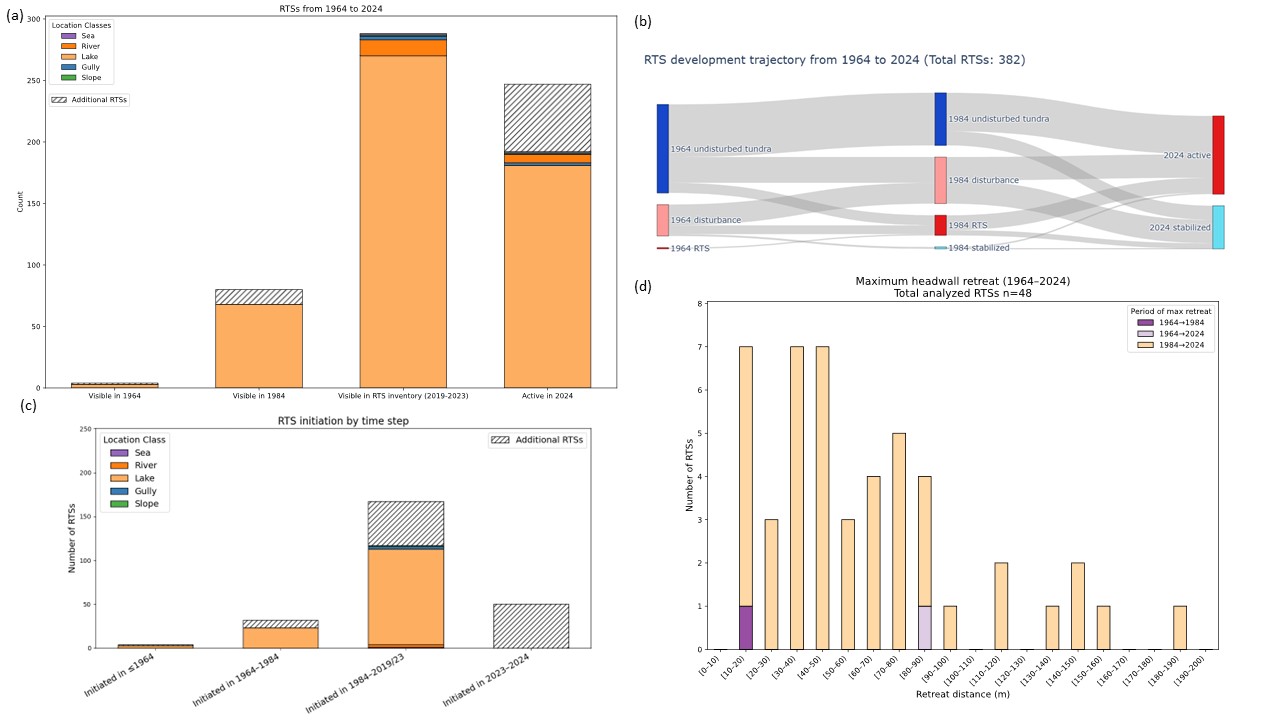
**

***Figure S-1-3: G2 key site RTS dynamics results: (a)*** *Counts of visible RTSs.* ***(b)*** *RTS development trajectory.* ***(c)*** *RTS initiation by time period.* ***(d)*** *Results of maximum headwall retreat color-coded by the time period of maximum retreat.*

**
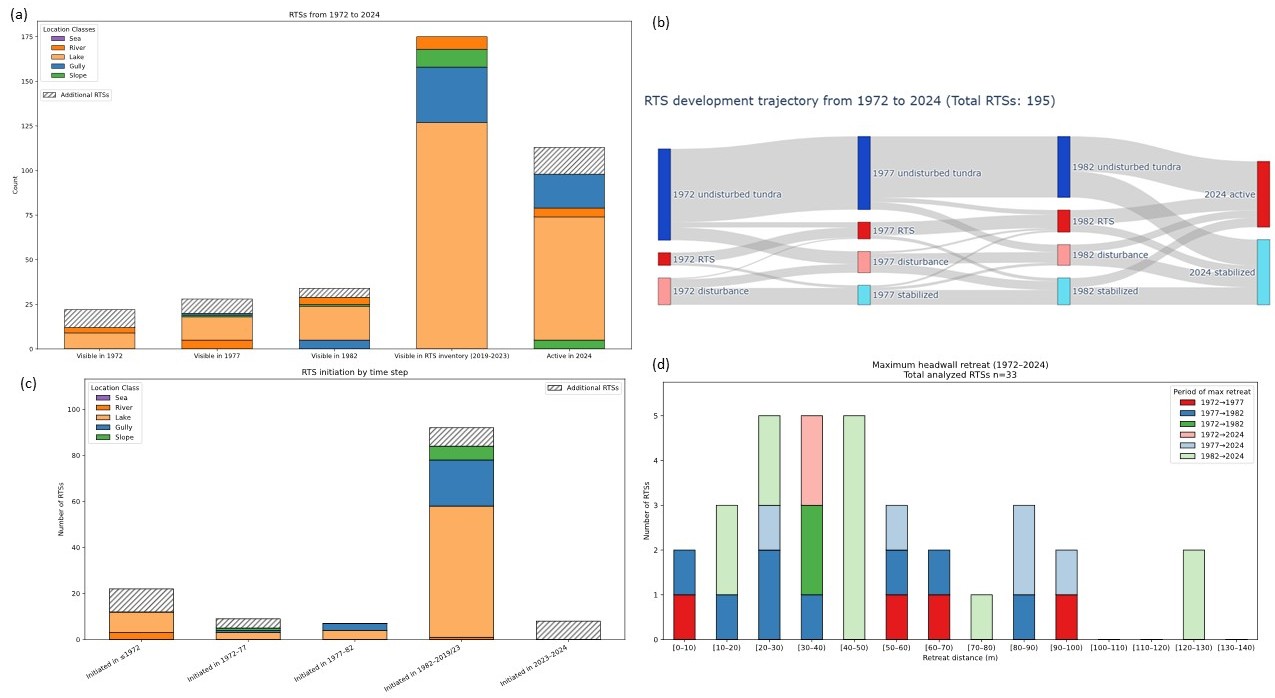
**

***Figure S-1-4: G3 key site RTS dynamics results: (a)*** *Counts of visible RTSs.* ***(b)*** *RTS development trajectory.* ***(c)*** *RTS initiation by time period.* ***(d)*** *Results of maximum headwall retreat color-coded by the time period of maximum retreat.*

*
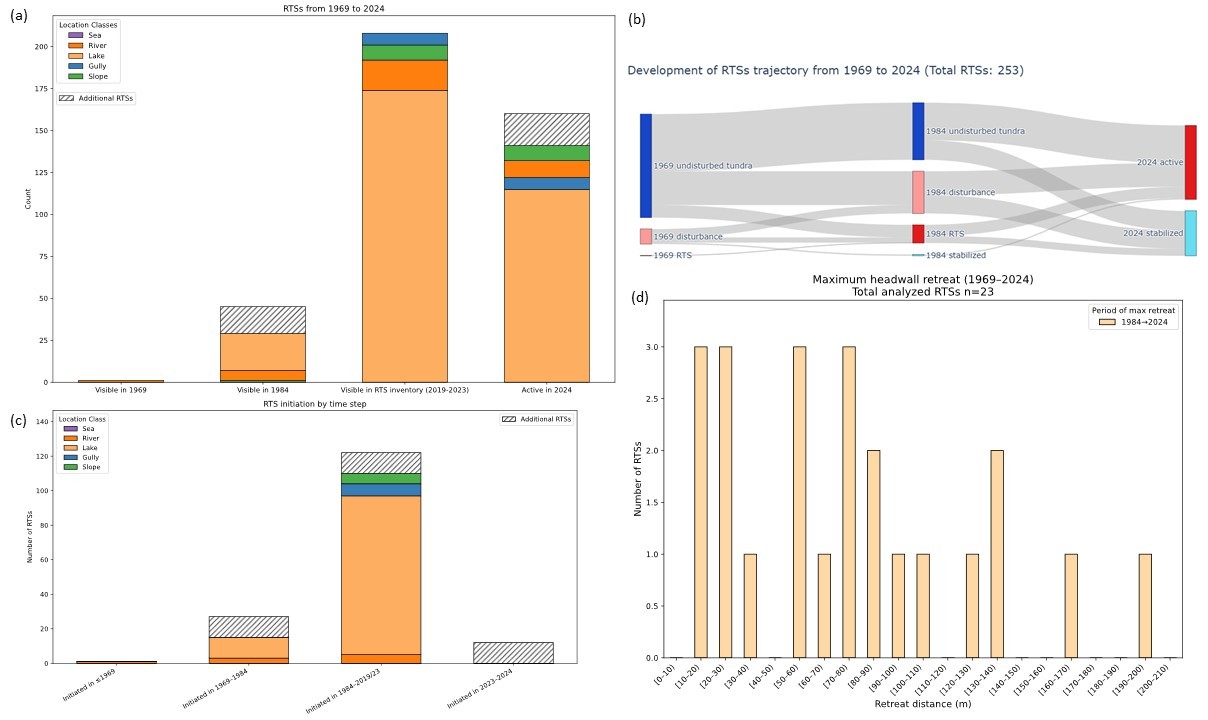
*

***Figure S-1-5: Y1 key site RTS dynamics results: (a)*** *Visible RTS count.* ***(b)*** *RTS development trajectory.* ***(c)*** *RTS initiation by time period.* ***(d)*** *Results of maximum headwall retreat color-coded by the time period of maximum retreat.*

***
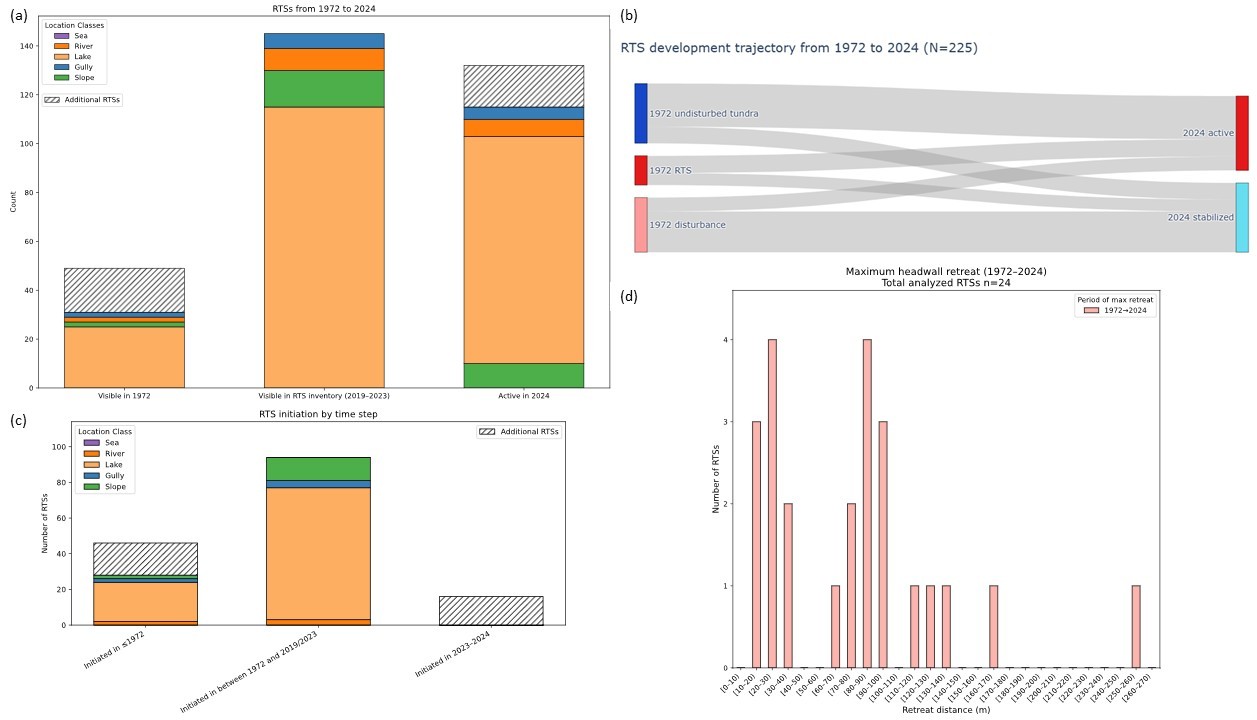
Figure S-1-6: Y2 key site RTS dynamics results: (a)*** *Counts of visible RTSs.* ***(b)*** *RTS development trajectory.* ***(c)*** *RTS initiation by time period.* ***(d)*** *Results of maximum headwall retreat color-coded by the time period of maximum retreat.*


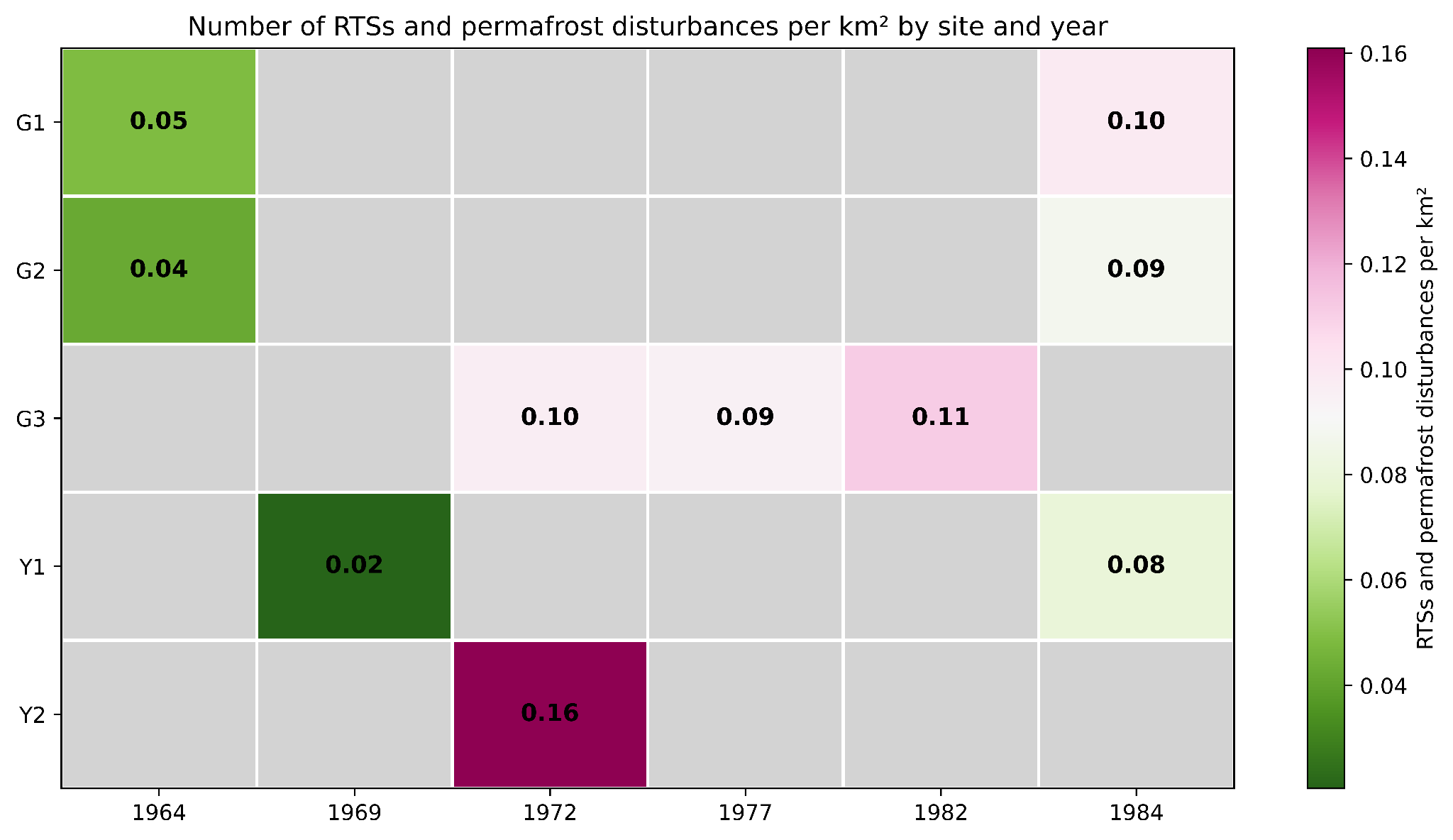


***Figure S-1-7:*** *Comparison of permafrost disturbances and RTSs standardized by area and year for all key sites.*

## 5. Bayesian climate hazard modelling of retrogressive thaw slump initiation – all model outcomes

Here, we present the results of the model outcomes that were not included in the main text of the article.

We studied 𝑆 = 1,947 sites across 𝑇 = 8 survey years (1964, 1969, 1972, 1977, 1982, 1984, 2019, 2024). Figure S-1-8 shows the stacked counts of presences, absences, and missing values per year. Early observations are sampled sparsely, while modern years approach complete coverage. We therefore relied on an absorbing survival likelihood that uses only pre-onset information and handles missingness explicitly.


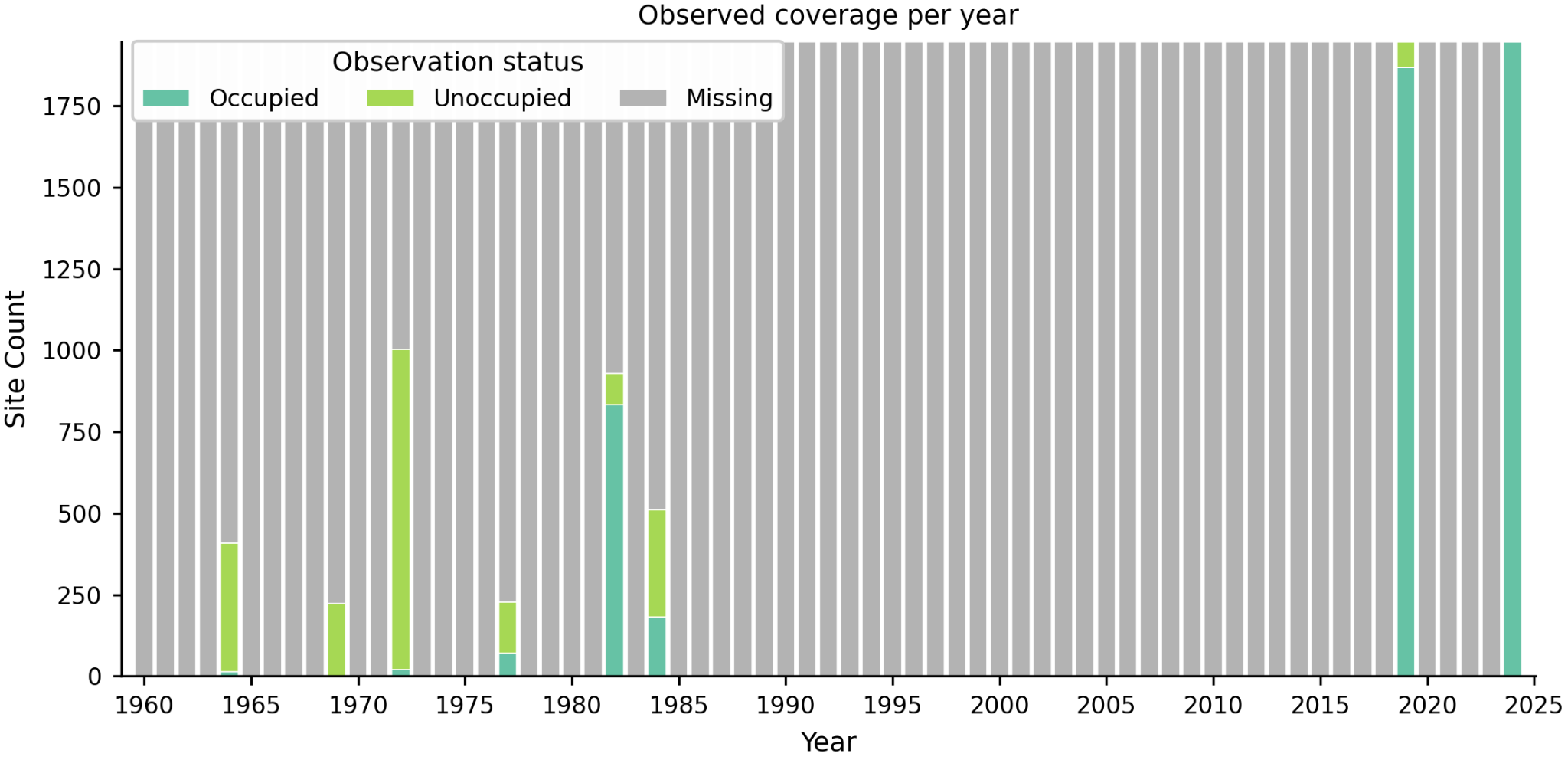


***Figure S-1-8.*** *Observed counts per year: occupied - the observed presence of RTSs (1), unoccupied - observed absence of RTS (0), and no observations (NaN). The figure shows sparse coverage in historical years and near-complete coverage in 2019–2024.*

Before fitting, we simulated absorbing occupancy paths from the prior distribution. Figure S-1-9 shows the prior-predictive distribution of cumulative occupied sites per year against observed totals among visited sites. The prior distribution admitted a gradual increase without forcing drastic growth, providing a reasonable baseline for learning from the data.

The model was fitted with 4 chains, 2,500 warmup, 2,500 draws. There were zero divergent transitions; all parameters showed R̂ ≤ 1.


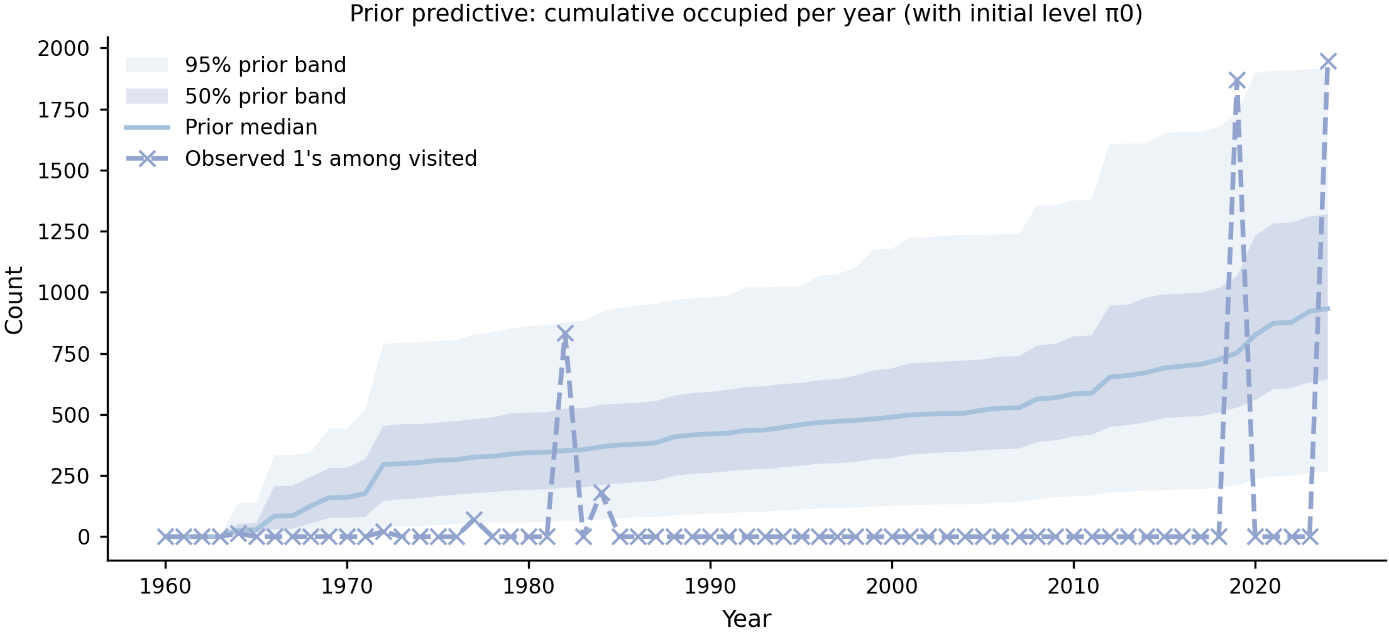


***Figure S-1-9.*** *Prior predictive totals for cumulative occupancy (median and 95% bands) with observed occupied counts. The prior allows plausible growth under the absorbing process.*

Relationships, while others covariates were fixed at empirical means, aligned with geomorphic expectations. Hazards increased steeply with summer maximum precipitation (sigmoidal; Figure S-1-10) and with winter mean temperature (Figure S-1-11). Summer mean temperature was uncertain (Figure S-1-12); winter maximum precipitation showed a modest positive trend with wide uncertainty (Figure S-1-13).

***
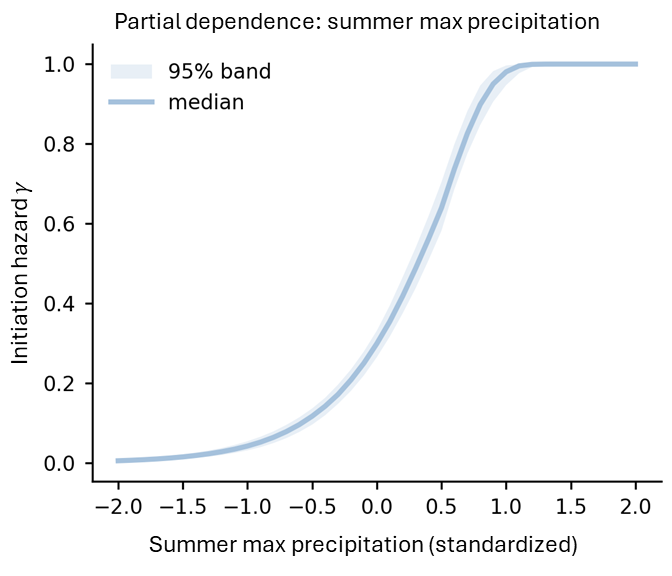
***

***Figure S-1-10.*** *Partial dependence of initiation hazard on summer maximum precipitation (standardized units). Pronounced, saturating increase. Bands are 95% posterior intervals.*

***
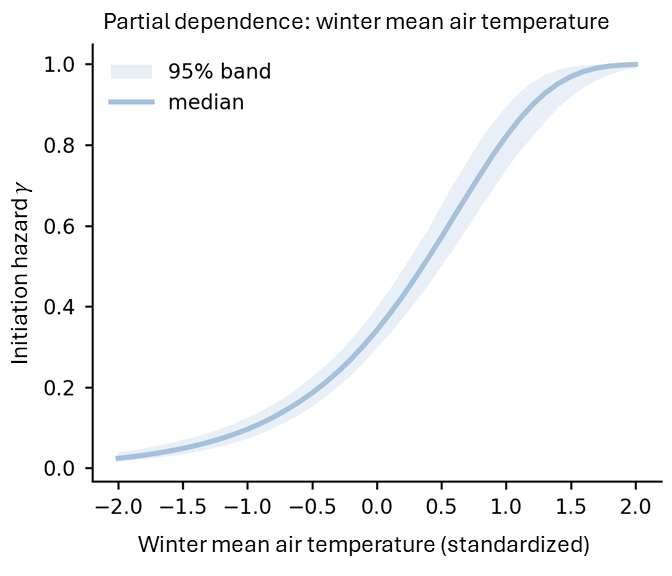
***

***Figure S-1-11.*** *Partial dependence on winter mean temperature (standardized units). Monotone increase consistent with preconditioning by warm winters.*

***
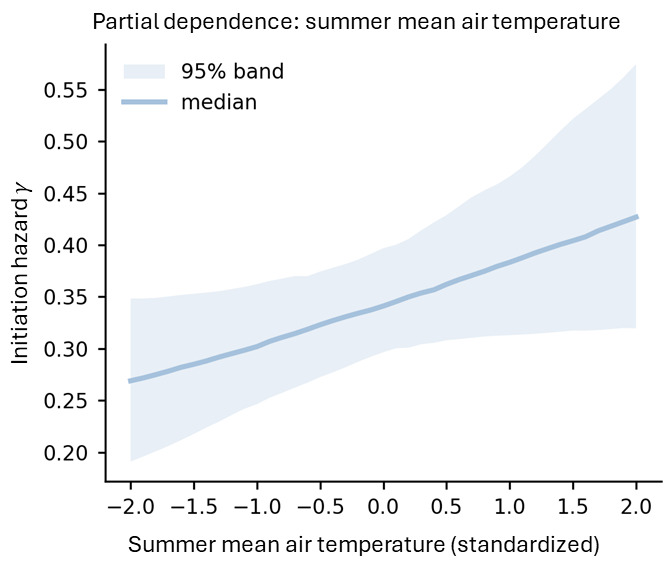
***

***Figure S-1-12.*** *Partial dependence on mean temperature in summer (standardized units). Weak relationship with wide uncertainty*.


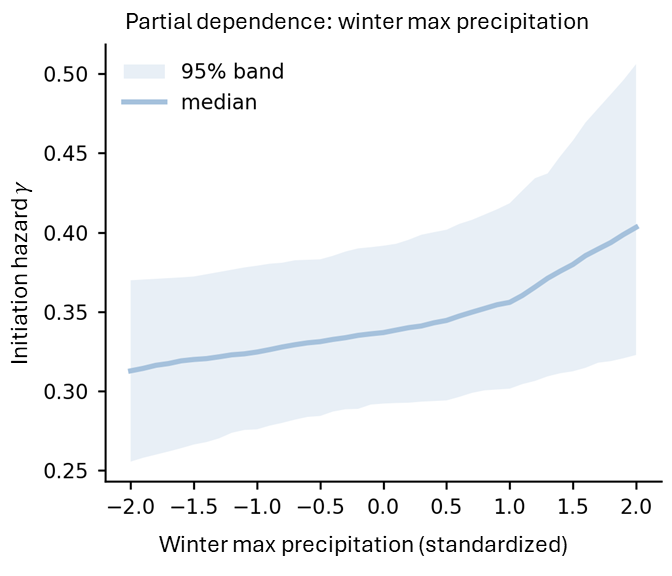


***Figure S-1-13.*** *Partial dependence on winter maximum precipitation (standardized units). Modest positive tendency.*

## 6. References

Generalov PP. 2000. State Geological Map of the Russian Federation (new series). Scale 1 : 1 000 000. Sheet R-(43)–45 (Gydan – Dudinka). Map of Pliocene-Quaternary formations.

Lopatyn BG. 2004. State Geological Map of the Russian Federation (new series). Scale 1 : 1 000 000. Sheet S-41–43 (Bely Island). Map of Pliocene-Quaternary formations.

Mardia KV, Jupp PE. 1999. Frontmatter. In: Directional Statistics. p 1–21 (Wiley Series in Probability and Statistics). [accessed 2025 Sept 10]. <https://doi.org/10.1002/9780470316979.fmatter>. <https://doi.org/10.1002/9780470316979.fmatter>

Massey FJ. 1951. The Kolmogorov-Smirnov Test for Goodness of Fit. Journal of the American Statistical Association. 46(253):68–78. <https://doi.org/10.2307/2280095>

Podsosova LL. 2000. State Geological Map of the Russian Federation (new series). Scale 1 : 1 000 000. Sheet R-(40)–42 (Vaigach Island–Yamal Peninsula). Map of Pliocene-Quaternary formations.

Pogrebitsky YuE, Musatov EE. 2000. State Geological Map of the Russian Federation (new series). Scale 1 : 1 000 000. Sheet S-44-46 (Ust-Tarea). Map of Quaternary formations.

Popov AI. 1985. Cryolithological map of the USSR (for permafrost area). Scale 1 .5: 4 000 000.

Trofimov VT. 1982. Map of genetic types and ice content of the upper 10-meter section of permafrost in the West Siberian Plate. Scale 1 : 1 000 000.

Zhdanov AV. 2014. State Geological Map of the Russian Federation, scale 1:1,000,000 (third generation). West Siberian series. Sheet Q-42 (Salekhard). Map of Pliocene-Quaternary formations.
