# Supplementary material for: Rapid increase in West Siberia’s retrogressive thaw slumps since 1964 associated with Arctic winter warming
Source: Sci Rep. 2026 Jun 11;16:18164. doi: 10.1038/s41598-026-56146-9 (PMC13254099; doi:10.1038/s41598-026-56146-9)
Supplement: Supplementary file 2 — Supplementary Information 2. [file 41598_2026_56146_MOESM2_ESM.docx]

**Supplementary Information 2**

**Extended Methods**

**for**

**Rapid increase in West Siberia’s retrogressive thaw slumps since 1964 associated with Arctic winter warming**

Nina Nesterova^1,2^, Marina Leibman^3^, Carl Stadie^1,4^, Tobias Hölzer^1,2^, Ingmar Nitze^1^, Ilia Tarasevich^5^, Kathrin Maier^6^, Maiia Vasileva^1,2^, Hugues Lantuit^1,2^, Jonas Küpper^1^, Guido Grosse^1,2^

1. Permafrost Research Section, Alfred Wegener Institute for Polar and Marine Research, 14473 Potsdam, Germany
2. Institute of Geosciences, University of Potsdam, 14469 Potsdam, Germany
3. Earth Cryosphere Institute, Tyumen Scientific Centre SB RAS, 625026, Tyumen, Russia
4. Faculty of Electrical Engineering and Computer Science, Technical University Berlin, 10623 Berlin, Germany
5. Department of Earth and Atmospheric Sciences, University of Houston, TX 77004, United States
6. Department of Environmental Engineering, ETH Zurich, 8093 Zurich, Switzerland

Correspondence to: Nina Nesterova, [nina.nesterova@awi.de](mailto:nina.nesterova@awi.de)

**Contents of this file**

Figures S2-1

Tables S2-1 - S2-2

# 1. Data collection and processing

## 1.1. Elevation

For elevation statistics, we aggregated elevation data from the ArcticDEM mosaics (Porter et al., 2023) version 4.1 with a 2 m resolution.

The elevation data were downloaded with the Python library Smart-Geocubes (Hölzer, 2025) and vertically reprojected to the NGA-EGM08 geoid as recommended by ArcticDEM guidelines for polar regions (NGA Geomatics, https://earth-info.nga.mil/index.php?dir=wgs84&action=wgs84).

The ArcticDEM validity mask and a waterbody mask (polygon layer, in detail explained in 1.2) were used to filter out potentially invalid data. For further analysis of mean elevation, we used the 95^th^ percentile of values for each H3 hexagon resolution 6, as well as for each key site to collect their elevation characteristics.

The vector ruggedness measure (VRM) is a measurement of terrain roughness based on the methodology developed by Riley et al. (1999) and Sappington et al. (2007). The metric is derived by expressing slope and aspect as 3-D vectors and, within a moving window, computing the magnitude of the resulting vector. For the VRM analysis per H3 hexagons, as well as for collecting key sites characteristics, we calculated the 95^th^ percentile of the VRM median values. We performed the VRM calculation in Python and followed the original ArcScripts implementation of the workflow (ESRI Community Water Resources Blog, 2020).

## 1.2. Waterbodies

To analyze waterbodies in the study area, we created vectors for *rivers* and *lakes* separately. The *river dataset* was created by combining river datasets from OpenStreetMap (OSM) (©OpenStreetMap contributors, ODbL 1.0, website: <https://www.openstreemap.org>, accessed 08.08.2025). Additionally, we used the OSM lakes database and manually selected rivers that were falsely classified as lakes.

To ensure the maximum coverage and minimum uncertainty, the lake dataset was created in several steps:

1. Based on the Global Surface Water dataset (Pekel et al., 2016), water bodies with the parameter *water occurrence* below 0.5 were removed. The remaining water bodies were then vectorized and filtered only for water bodies larger than 1500 m^2^ to remove temporary water accumulation and false positives.

2. The resulting waterbody polygons were masked by the *river dataset* to filter out the rivers.

3. Missing lakes were added from the OSM lake dataset, as well as lakes falsely classified as rivers from the OSM river dataset. This false-positive filtering of the OSM river and lake dataset was performed manually.

4. To minimize false positives in the resulting lake polygons, they were masked by the vectorized infrastructure dataset SACHI_v2 (Bartsch et al., 2021)

## 1.3. Landcover

We analyzed the landcover for the Yamal and Gydan peninsulas using a new circumarctic land cover classification that takes into account Arctic vegetation types and wetness gradients (Bartsch et al., 2024). The missing piece of South-Western Gydan was processed by the authors of this work upon our request. The dataset was clipped to the study area and reprojected to WGS 84/ UTM Zone 43 (EPSG:32643).

## 1.4. Infrastructure

To analyze the infrastructure, we used the OSM dataset (©OpenStreetMap contributors, ODbL 1.0, website: <https://www.openstreemap.org>, accessed 08.08.2025). We downloaded linear and polygonal vectors using a query request at [www.overpass-turbo.eu](http://www.overpass-turbo.eu). We have classified infrastructure into high-, medium-, and low-criticality categories based on the European Environmental Agency's conceptual distinction (European Environment Agency, 2024). The infrastructure vectors for the following categories were downloaded:

1. High criticality infrastructure
2. Energy: Oil & Gas (pipelines + pipeline stations/manifolds; no markers/valves); Electricity backbone (transmission + substations + plants)
3. Health: Hospitals
4. Transport: Aviation (aerodromes/runways/taxiways/terminals); Rail (railways); Bridges (only bridges that carry trunk/primary/secondary roads or main rail); Ports (harbour/port landuse + facilities)
5. Medium criticality infrastructure
6. Transport: secondary highways
7. Energy: Minor powerlines
8. Water: Engineered water management
9. Logistics: Warehousing/depots/rail yard landuse
10. Low criticality infrastructure
11. Transport/Local access: local roads, minor bridges

## 1.5. Climate data

Temperature and precipitation data were obtained from the ERA5‑Land reanalysis dataset via the Destination Earth “Earth Data Hub” (Hersbach et al., 2020).

ERA5‑Land provides hourly fields on a 0.1° regular grid. To analyse seasonal climate characteristics, the raw hourly data were first aggregated temporally and spatially to generate yearly summer and winter summaries for each study region. The summer season was defined as May - September, whereas the winter season comprised October - December of the preceding year, together with January - April of the current year. 2‑m air temperature values were converted to daily means by averaging the daily minimum and maximum temperatures, following the methodology described by the Government of Canada (accessed 01 September 2025).

From these daily mean temperatures, the following thaw‑relevant metrics were derived for each year: Thawing degree‑days (° · day), Number of thawing days (days), Date of the first thawing day (calendar day), Date of the last thawing day (calendar day), Duration of the thawing season (days).

For each region, the daily mean temperatures were subsequently averaged over all grid cells within the region and over the entire season, yielding two seasonal temperature time series (summer and winter) expressed in Kelvin (K).

Hourly precipitation accumulations were summed to obtain total precipitation for each 0.1° grid cell over the defined summer and winter periods. Within each region, the seasonal totals were then reduced to a single representative value using the (mean – for plotting Figure 4, maximum - for Bayesian modelling). The resulting seasonal precipitation series are expressed as depth equivalents (m), equivalent to volume per unit area (m³ · m⁻²).

## 1.6. RTS polygons 1964-1969

To map RTS polygons for 1964-1969, we georeferenced and analyzed historical CORONA imagery (1964-1969) (Grosse et al., 2005).

### Georeferencing

Image georeferencing was performed in the open-source software QGIS version 3.34.8, using the ESRI Satellite basemap as a reference layer. We used a 3rd polynomial transformation method (Mohammed et al., 2013). The number of ground control points (GCPs) varied depending on terrain characteristics, image properties, and the mean error (RMS) reported by the QGIS georeferencing module, but was not less than 40 points for each image (Table S-2 - 1). As the resampling method, we used a nearest neighbour algorithm.

### Mapping

For delineating RTSs, we used the QGIS Geo-SAM plugin (Zhao et al., 2023), which is based on the Segment Anything Model (SAM). Before segmentation, each image needs to be encoded with the built-in algorithm of the plugin. After encoding, segmentation can be performed by selecting the relevant image and defining (or selecting an existing) vector layer into which the segmented objects will be stored. Within this study, the Geo-SAM plugin demonstrated high applicability: the majority of clearly distinguishable RTSs visible on historical imagery were effectively extracted. Nevertheless, in cases where RTS could not be identified on the image clearly, or the plugin produced suboptimal results, we manually delineated the RTS instances.

### Classification

During the delineation process, we created a classification system for the segmented objects. All objects were assigned to one of nine classes with corresponding numerical codes:

0 – RTS, visually clearly identified RTS with no doubts

1 – disturbance feature, uncertain on the type of permafrost disturbance

2 – undisturbed tundra

3 – delineation problem (e.g., object located at image edge or obscured by clouds)

6 – ALDs (Active layer detachment slides, explained in detail in 1.7)

9 – stabilized disturbance (explained in detail in 1.7)

40 – RTS, visually clearly identified RTS with no doubts, mapped additionally to the inventory dataset (explained in detail in 1.7)

41 – disturbance feature, mapped additionally to the inventory dataset (explained in detail in 1.7)

42 – stabilized disturbance (explained in detail in 1.7)

43 – undisturbed tundra, mapped additionally to the inventory dataset (explained in detail in 1.7)

Class 0 was assigned to objects that could be visually identified as RTS, for example, when the headwall was clearly visible in the image. Class 1 was used when surface disturbance was evident, but it was not possible to confidently determine whether it represented an RTS or another type of disturbance. Class 2 objects were created when no surface disturbance was observed at the location of an inventory point in the given image. Class 3 was used when a visibility issue occurred at the location of an inventory point, such as cloud cover or when the point was located near the image edge, where severe distortion was present. The confidence level included three categories: 1 – confident, 2 – uncertain, and 3 – no data / no confidence.

The example of mapped and classified RTS is demonstrated in Figure S-2 -1.

**Table S-2 – 1. Metadata and georefencing uncertainty for CORONA images**

| Key site | File name | Source | Acquisition date (DD.MM.YYYY) | Base image | GCPs | RMS | Method | Resampling | Pixelsize |
| --- | --- | --- | --- | --- | --- | --- | --- | --- | --- |
| G1 | DS1009-1006DF010_b | CORONA | 06.08.1964 | ESRI basemap | 44 | 2.7 | 3rd polynomial | nearest neighbour | 2.75 m |
| G2 | DS1009-1006DA021_b | CORONA | 06.08.1964 | ESRI basemap | 59 | 2.5 | 3rd polynomial | nearest neighbour | 2.75 m |
|  | DS1009-1006DA021_c | CORONA | 06.08.1964 | ESRI basemap | 126 | 3 | 3rd polynomial | nearest neighbour | 2.75 m |
| Y1 | DS1052-1088DF001_b | CORONA | 06.08.1964 | ESRI basemap | 75 | 3.1 | 3rd polynomial | nearest neighbour | 2.75 m |
|  | DS1052-1088DF002_b | CORONA | 06.08.1964 | ESRI basemap | 104 | 3.1 | 3rd polynomial | nearest neighbour | 2.75 m |

## 1.7. RTS polygons 1972-1984

To map RTS polygons for 1972-1984, we georeferenced and analyzed historical HEXAGON imagery (1972-1984) (Hammer et al., 2022).

### Georeferencing

HEXAGON imagery was also georeferenced in QGIS, following the same procedure as for CORONA imagery, with the ESRI Satellite basemap used as reference. We also used a 3rd *polynomial transformation* (1) with nearest neighbour resampling (Table S-2 - 2). However, in certain cases, we used the *Thin Plate Spline* method with cubic (4x4 kernel) resampling (2) instead. The choice of method depended on image-specific characteristics and was based on a visual assessment of the georeferencing quality: method (1) was suitable for most images, as they were of high quality and acquired in (near-) nadir orientation. For images acquired at an oblique angle or experiencing distortions, method (2) performed better. For each image, around 40-50 Ground Control Points (GCPs) were created.

### Mapping

For the RTS segmentation, we used the Geo-SAM plugin where possible and supplemented it by manual delineation when the plugin failed to produce accurate results or when the object could not be automatically recognized. A key challenge during this process was the heterogeneity in radiometric image characteristics, particularly image brightness. Whereas CORONA imagery exhibited relatively uniform brightness levels, HEXAGON image brightness varied substantially across acquisition dates, which made their comparison within the same key site more challenging. Although these issues could be partially mitigated by adjusting image histogram parameters (e.g., brightness/contrast), it was not possible to achieve uniform visual quality across all images. While this did not significantly affect final results, it introduced challenges for the automated digitization process.

### Classification

During the delineation of RTS, we noticed widespread ALDs in some key sites. These features were recognizable due to their elongated shape and “thread-like” structure compared to other types of surface disturbance. We decided to separate them into a distinct class (Class 6). However, they were not included in the further analysis, as no clear relationship between ALD and RTS was identified; in other words, RTS initiation was not strongly linked to ALD sites.

Since the HEXAGON images represent a later observation period than CORONA, and for some key sites, both datasets are available, they correspond to the second stage of the analysis. During digitization, we added a new class to the classification scheme – class 42 – stabilized feature, mapped additionally to the inventory dataset. This class was assigned only to “new” RTS, i.e., those not included in the initial inventory dataset. It was used when an RTS was present in the earlier period (i.e., on CORONA imagery or on an earlier HEXAGON image), but was no longer visible in the later period, which indicated that the feature had stabilized by that time.

To enable more accurate comparison of delineated objects across different years, we introduced an additional class: 43 – undisturbed tundra, mapped additionally to the inventory dataset. Objects in this class were added to the dataset only after segmentation for all periods on a given site was completed. This class was assigned when a new RTS, not included in the inventory, was detected on later imagery but absent on earlier imagery.

In addition, we introduced class 9 – stabilized disturbance (within the inventory dataset). This class has the same meaning as class 42, but it applies to RTS recorded in the initial inventory dataset.

We also created and followed logical rules for classification:

• If an object had been classified as 0 (RTS, visually clearly identified RTS with no doubts) or 1 (disturbance of uncertain type) in the earlier period, it could not be reclassified as 2 (undisturbed tundra) in the later period.

• Similarly, an object classified as 0 (RTS, visually clearly identified RTS with no doubts) in the earlier period could not logically be downgraded to 1 (disturbance feature, uncertain on the type of permafrost disturbance) in the later period. However, due to the aforementioned image quality differences, in some cases, accurate delineation was indeed more difficult in the later imagery. To resolve this issue and avoid logical inconsistencies, a confidence level was introduced for objects in class 0, indicating the graphical precision of the delineation. The confidence level included three categories: 1 – confident, 2 – uncertain, and 3 – no data / no confidence.

The example of mapped and classified RTS is demonstrated in Figure S-2 -1.

**Table S-2 – 2. Metadata and georeferencing error table for HEXAGON images.**

| Key site | File name | Source | Acquisition date  DD.MM.YYYY | Base image | GCPs | RMS | Method | Resampling | Pixel size |
| --- | --- | --- | --- | --- | --- | --- | --- | --- | --- |
| G1 | D3C1219-100275F007_a | HEXAGON | 29.07.1984 | ESRI basemap | 43 | 4.4 | 3rd polynomial | nearest neighbor | 1m |
|  | D3C1219-100275F007_b | HEXAGON | 29.07.1984 | ESRI basemap | 41 | 4.7 | 3rd polynomial | nearest neighbor | 1m |
|  | D3C1219-100275F008_a | HEXAGON | 29.07.1984 | ESRI basemap | 41 | 3.5 | 3rd polynomial | nearest neighbor | 1m |
|  | D3C1219-100275F009_a | HEXAGON | 29.07.1984 | ESRI basemap | 44 | 4.6 | 3rd polynomial | nearest neighbor | 1m |
|  | D3C1219-100275F006_b | HEXAGON | 29.07.1984 | ESRI basemap | 41 | 4.4 | 3rd polynomial | nearest neighbor | 1m |
| G2 | D3C1219-100275F009_d | HEXAGON | 29.07.1984 | ESRI basemap | 42 | 4.1 | 3rd polynomial | nearest neighbor | 1m |
|  | D3C1219-100275F010_d | HEXAGON | 29.07.1984 | ESRI basemap | 44 | 3.4 | 3rd polynomial | nearest neighbor | 1m |
|  | D3C1219-100275F011_c | HEXAGON | 29.07.1984 | ESRI basemap | 44 | 4.0 | 3rd polynomial | nearest neighbor | 1m |
|  | D3C1219-100275F011_d | HEXAGON | 29.07.1984 | ESRI basemap | 38 | 3.4 | 3rd polynomial | nearest neighbor | 1m |
|  | D3C1219-100275F012_c | HEXAGON | 29.07.1984 | ESRI basemap | 40 | 4.6 | 3rd polynomial | nearest neighbor | 1m |
|  | D3C1219-100275F008_d | HEXAGON | 29.07.1984 | ESRI basemap | 43 | 3.5 | 3rd polynomial | nearest neighbor | 1m |
|  | D3C1219-100275F008_e | HEXAGON | 29.07.1984 | ESRI basemap | 27 | 3.6 | 3rd polynomial | nearest neighbor | 1m |
|  | D3C1219-100275F009e | HEXAGON | 29.07.1984 | ESRI basemap | 37 | 4.1 | 3rd polynomial | nearest neighbor | 1m |
|  | D3C1219-100275F010_c | HEXAGON | 29.07.1984 | ESRI basemap | 42 | 4.8 | 3rd polynomial | nearest neighbor | 1m |
| G3 | D3C1203-400576A020_b | HEXAGON | 22.08.1972 | ESRI basemap | 71 | 4.5 | 3rd polynomial | nearest neighbor | 1m |


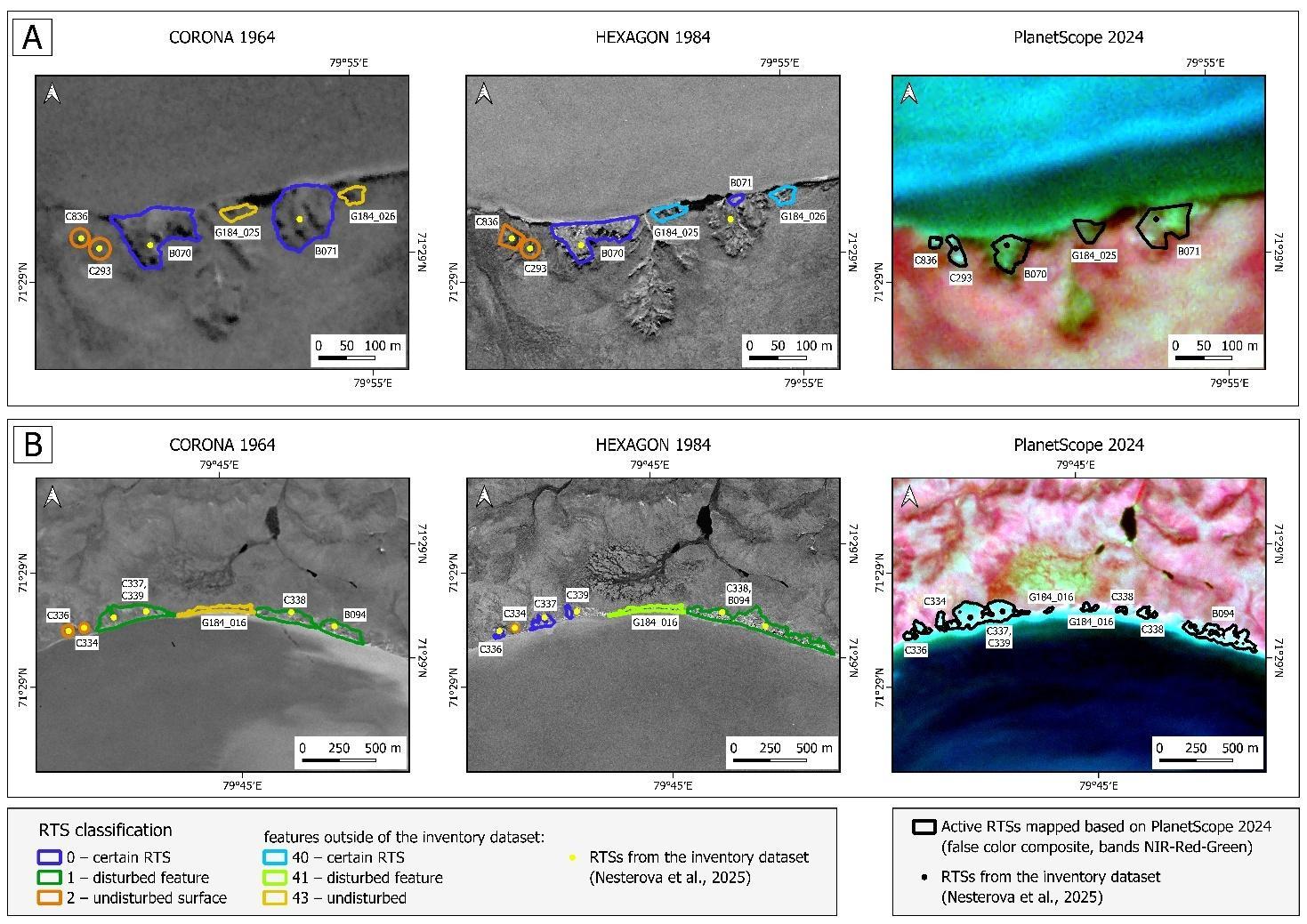


***Figure S-2- 1: The examples of RTS polygon delineation and classification on historical CORONA and HEXAGON imagery and modern PlanetScope imagery. Projection: WGS 84/ UTM Zone 43. The map was created using QGIS Desktop v. 3.36.2 (see Methods).***

## 1.8. RTS polygons 2024

To create RTS polygons for 2024, we developed a semi-automated method. First, we used PlanetScope 2024 Surface Reflectance Imagery (Planet Team, 2018) in False Color Composite Red combination (NIR, Red, Green) to map active RTSs in the Y2 key site. We delineated the grey-blue outlines of RTSs (Figure S2-2). Then we applied an AI-based automated RTS detection model, DARTS, that uses high-resolution remote sensing and Unet++ Convolutional Neural Networks (Nitze et al., 2025). We trained this model on the Y2 polygons and rerun it for G1, G2, G3, and Y1 key sites. Finally, we manually filtered the resulting RTS areas and removed false positives, then we manually delineated the missing ones.

## 1.9. General RTS polygon naming

We developed a naming convention: RTS polygons of the inventory (Nesterova et al., 2025) inherited their identifier (ID). For additionally mapped RTSs, the naming followed the logic of “key site name_year first observed_sequence number”. For example, if RTS was first observed in Y1 in 1984, it is “Y184_...” or if RTS is first observed in 2024 in G1, it is “G124_...”.

## 1.10. Headwall retreat estimations

For estimating headwall retreat, we created a Python code that iteratively cycled through growing 1 m buffers starting with the earliest year RTS polygon (i.e., 1964) until it covered the latest year polygon (i.e., 1984). The radius of the resulting buffer was considered as a first approximation of headwall retreat. This rather simple but easy-to-automate method demonstrated robust results when compared to manually measured headwall retreat in QGIS software. The median RMSE of 4.1 m found during the georeferencing (Tables S-2-1 and S-2-2) was applied for each year to calculate headwall retreat uncertainty. The bars indicating headwall retreat uncertainty are shown in Figure 3 of the main text.

## 1.11. Initiation rates

The initiation rate was estimated as the number of new RTS per year. We have not normalized initiation rates per area due to the arbitrary key site boundaries that were selected based on data availability. Thus, key sites may include areas with no RTSs at all. Then, normalization by their areas can be misleading.

## 1.12. Tiering the infrastructure by risk

To classify infrastructure by the risk of RTS further development, we have set up the following five tiers:

1. Tier 1, Critical risk

This class includes all the infrastructure of a high criticality that is currently exposed (overlaps with current RTS clusters) or the infrastructure that is located within a 300 m vicinity of the nearest RTS. The distance of 300 m was selected based on the median RTS area of 2.5 ha found in Leibman et al. (2023) study and an additional buffer to account for RTS being a point in the dataset of Nesterova et al. (2025).

1. Tier 2, High risk

This class includes all the infrastructure of a medium criticality that is currently exposed (overlaps with current RTS clusters) or all the infrastructure of a high criticality that is potentially at risk (overlaps with potential RTS clusters).

1. Tier 3, Medium risk

This class includes all the infrastructure of a low criticality that is currently exposed (overlaps with current RTS clusters) or all the infrastructure of a medium criticality that is potentially at risk (overlaps with potential RTS clusters).

1. Tier 4, Low risk

This class includes all the infrastructure of a low criticality that is potentially at risk (overlaps with potential RTS clusters) or any infrastructure that is in the same hexagonal H3 grid cell with at least one RTS present.

1. Tier 5, Very low risk

This class includes all the infrastructure that is in the hexagonal H3 grid cells with no RTS present.

## 1.13. Retrieval of climate data by scenario

To collect predicted air temperature change by 2100, we have used the “CMIP6 mean” scenario ensemble data available via the KNMI Climate Explorer (2022) (van der Wiel, 2024). “CMIP6 mean” represents the mean of a subset of 33 CMIP6 models, where for each model multiple runs were averaged. The coordinates selected correspond to the bounding box of all key sites. The predicted climate variables differ according to the Shared Socioeconomic Pathways (SSPs: 1-2.6, 2-4.5, 3-7.0. 5-8.5) and emission scenarios (Tebaldi et al., 2021). The resulting air temperature change was aggregated to represent a year's winter season air temperature encompassing October-December of the preceding year and January-April of the current year [year in question, data year, modelled year, designated year].

# 2. Bayesian climate hazard modelling of retrogressive thaw slump initiation

## Overview

We modeled RTS initiation only, and once an RTS was detected at a site, it was treated as permanently present for initiation risk assessment (an absorbing state; no “turning off” is modeled) (Kokelj et al., 2015). Year-to-year changes in risk were assumed to come only from climate covariates. We did not add an unconstrained year-to-year term to model unexplained variation between years, to prevent this baseline term from absorbing climate-driven trends (Allison, 1982). To summarize effect sizes on an interpretable scale, we compute average marginal effects (AMEs) over the risk set, the set of site–year cells that are still uninitiated at that time (pre-onset only).

## Data preparation and variable selection

We assembled a site (𝑖 = 1, . . . , 𝑆) by year (𝑡 = 1, . . . , 𝑇) matrix 𝑌 = {𝑦𝑖𝑡 } where every site (RTS) at every given time was noted as either present (1), absent (0), or not observed (NA). To reduce redundancy among climate covariates, we used a principal component analysis (PCA), which indicated three interpretable modes: (i) a winter preconditioning axis with strong positive loadings for winter mean temperature and winter precipitation; (ii) a summer rainfall intensity axis dominated by summer maximum precipitation; and (iii) a weaker summer thermal background axis aligned with summer mean temperature. Based on this, we kept four predictors that span these modes with minimal redundancy: winter mean temperature and winter maximum precipitation as representatives of the winter preconditioning axis; summer maximum precipitation as the rainfall–trigger proxy; and summer mean temperature to capture the thaw–season background.

We aligned the four covariates to the site–year matrix and standardized each to zero mean and unit variance over all sites–years, to place coefficients on a comparable scale and stabilize Hamiltonian Monte Carlo (Betancourt, 2018). To represent multi-year winter preconditioning, we added residualized exponential moving-average (EWMA) features for winter mean temperature and winter maximum precipitation.

To allow accelerating risk at higher values, consistent with threshold-like thermo-erosion/runoff behaviour, we added hinge terms ℎ_𝑧_ (𝑥^∗^) = max(0, 𝑥^∗^ − 𝑧) at standardized knots 𝑧 ∈ {0.5, 1.0} (Friedman, 1991; Wood, 2017). We further ensured the effects of covariates on RTS initiation were non-negative via HalfNormal priors.

## RTS Initiation Hazard Model

### Model Setup

For uninitiated sites, the annual initiation hazard is modelled as

𝛾_𝑖𝑡_ = Pr(𝑧_𝑖,𝑡_ = 1 | 𝑧_𝑖,𝑡_−1 = 0, x_𝑖𝑡_ ),

with 𝑧𝑖𝑡 ∈ {0, 1} indicating whether the site has initiated by the end of year 𝑡. We use a complementary log–log (cloglog) link,

cloglog(𝛾_𝑖𝑡_ ) = 𝛽_0,𝛾_ + 𝑎_𝑖_ + 𝑢_𝑡_ + x^∗⊤^ _𝑖𝑡_ B_𝛾_, 𝛾_𝑖𝑡_ = 1 − exp{− exp(·)},

which is the discrete-time proportional-hazards form (Prentice and Gloeckler, 1978; Allison, 1982). Persistent site susceptibility (e.g., substrate, relief) is captured by the site frailty 𝑎_𝑖_ ∼ *N* (0, 𝜎^2^ _site_).

To separate early detections attributable to pre-existing initiation from first-year hazards, we include a scalar 𝜋_0_ ∼ Beta (1, 9), the fraction already initiated at each site’s *first observed year*. If the first observation is 1, the contribution is log {𝜋_0_ + (1 − 𝜋_0_) 𝛾𝑖𝑡 }; if it is 0, the contribution is log{(1 − 𝜋_0_) (1 − 𝛾_𝑖𝑡_ )}. After the first observed year, contributions follow the standard absorbing survival form.

We use weakly informative priors: 𝛽_0,𝛾_ ∼ *N*(log 0.002, 0.8^2^), 𝜎_site_ ∼ HalfNormal(0.25); slope priors are HalfNormal. We fit with chains, tune=2500, draws=2500, target_accept=0.995), which proved stable for our design (Betancourt, 2017). Prior-predictive simulations propagate hazards through absorbing paths (including 𝜋_0_) to ensure plausibility before conditioning on data.

After sampling, we computed (i) hazard ratios exp{Δ𝐵_𝛾,𝑘_ } for a standardized change Δ e.g, a multiplicative change in the annual chance of first initiation for an uninitiated site, and (ii) percentage-point changes in 𝛾𝑖𝑡 averaged over empirical site–year rows from the risk set only. We compute Savage–Dickey Bayes factors using the constrained vs. unconstrained prior density at zero and a KDE estimate of the posterior density at zero; the prior density at zero differs for HalfNormal and Normal priors.

### Diagnostics and predictive checks

We assessed sampler health with $\hat{R}$, Bulk/tail ESS, and divergences. Posterior-predictive checks simulate full absorbing initiation paths under posterior hazards and compare *new onsets per year* to observed first detections (Allison, 1982). We also compare 𝛾𝑖𝑡 at observed pre-onset zeros versus onset years, and produce partial-dependence curves (hazard vs. one standardized covariate at a time, with others fixed at empirical means) to visualize the monotone/threshold patterns implied by precipitation hinges (Friedman, 1991; Wood, 2017).

# 3. References

Allison, P. D.: Discrete-Time Methods for the Analysis of Event Histories, Sociological Methodology, 13, 61–98, <https://doi.org/10.2307/270718>, 1982.

Andrew Gelman, Aleks Jakulin, Maria Grazia Pittau, and Yu-Sung Su: A weakly informative default prior distribution for logistic and other regression models, The Annals of Applied Statistics, 2, 1360–1383, <https://doi.org/10.1214/08-AOAS191>, 2008.

NGA Geomatics - WGS 84: <https://earth-info.nga.mil/index.php?dir=wgs84&action=wgs84>, last access: 9 September 2025.

OpenStreetMap: <https://www.openstreetmap.org/>, last access: 8 August 2025.

Bartsch, A., Pointner, G., Nitze, I., Efimova, A., Jakober, D., Ley, S., Högström, E., Grosse, G., and Schweitzer, P.: Expanding infrastructure and growing anthropogenic impacts along Arctic coasts, Environmental Research Letters, 16, 115013, <https://doi.org/10.1088/1748-9326/ac3176>, 2021.

Bartsch, A., Efimova, A., Widhalm, B., Muri, X., von Baeckmann, C., Bergstedt, H., Ermokhina, K., Hugelius, G., Heim, B., and Leibman, M.: Circumarctic land cover diversity considering wetness gradients, Hydrol. Earth Syst. Sci., 28, 2421–2481, <https://doi.org/10.5194/hess-28-2421-2024>, 2024.

Betancourt, M.: A Conceptual Introduction to Hamiltonian Monte Carlo, <https://doi.org/10.48550/arXiv.1701.02434>, 16 July 2018.

KNMI Climate Explorer: <https://climexp.knmi.nl/start.cgi>, last access: 10 February 2026.

Terrain Ruggedness Index (TRI) and Vector Ruggedness Measurement (VRM) - Two new Arc Hydro functions that quantify ruggedness on a DEM: <https://community.esri.com/t5/water-resources-blog/terrain-ruggedness-index-tri-and-vector-ruggedness/ba-p/884340>, last access: 9 September 2025.

European Environment Agency: European climate risk assessment, Publications Office of the European Union, <https://doi.org/10.2800/8671471>, 2024.

Feng, L., Smith, S. J., Braun, C., Crippa, M., Gidden, M. J., Hoesly, R., Klimont, Z., van Marle, M., van den Berg, M., and van der Werf, G. R.: The generation of gridded emissions data for CMIP6, Geoscientific Model Development, 13, 461–482, <https://doi.org/10.5194/gmd-13-461-2020>, 2020.

Friedman, J. H.: Multivariate Adaptive Regression Splines, The Annals of Statistics, 19, 1–67, <https://doi.org/10.1214/aos/1176347963>, 1991.

Glossary - Climate - Environment and Climate Change Canada: <https://climate.weather.gc.ca/glossary_e.html#climatological_day>, last access: 9 September 2025.

Grosse, G., Schirrmeister, L., Kunitsky, V. V., and Hubberten, H.-W.: The use of CORONA images in remote sensing of periglacial geomorphology: an illustration from the NE Siberian coast, Permafrost and Periglacial Processes, 16, 163–172, <https://doi.org/10.1002/ppp.509>, 2005.

Hammer, E., FitzPatrick, M., and Ur, J.: Succeeding CORONA: declassified HEXAGON intelligence imagery for archaeological and historical research, Antiquity, 96, 679–695, <https://doi.org/10.15184/aqy.2022.22>, 2022.

Hersbach, H., Bell, B., Berrisford, P., Hirahara, S., Horányi, A., Muñoz-Sabater, J., Nicolas, J., Peubey, C., Radu, R., Schepers, D., Simmons, A., Soci, C., Abdalla, S., Abellan, X., Balsamo, G., Bechtold, P., Biavati, G., Bidlot, J., Bonavita, M., De Chiara, G., Dahlgren, P., Dee, D., Diamantakis, M., Dragani, R., Flemming, J., Forbes, R., Fuentes, M., Geer, A., Haimberger, L., Healy, S., Hogan, R. J., Hólm, E., Janisková, M., Keeley, S., Laloyaux, P., Lopez, P., Lupu, C., Radnoti, G., de Rosnay, P., Rozum, I., Vamborg, F., Villaume, S., and Thépaut, J.-N.: The ERA5 global reanalysis, Quarterly Journal of the Royal Meteorological Society, 146, 1999–2049, <https://doi.org/10.1002/qj.3803>, 2020.

Hölzer, T.: relativityhd/smart-geocubes: Release v0.0.8, , <https://doi.org/10.5281/zenodo.15232755>, 2025.

Kokelj, S. V., Tunnicliffe, J., Lacelle, D., Lantz, T. C., Chin, K. S., and Fraser, R.: Increased precipitation drives mega slump development and destabilization of ice-rich permafrost terrain, northwestern Canada, Global and Planetary Change, 129, 56–68, <https://doi.org/10.1016/j.gloplacha.2015.02.008>, 2015.

Leibman, M., Nesterova, N., and Altukhov, M.: Distribution and Morphometry of Thermocirques in the North of West Siberia, Russia, Geosciences, 13, <https://doi.org/10.3390/geosciences13060167>, 2023.

Mohammed, N. Z., Eisa, E., and Elhaj, A.: The effect of polynomial order on georeferencing remote sensing images, International Journal of Engineering and Innovative Technology (IJEIT), 2, 5–8, 2013.

Nesterova, N., Tarasevich, I., Leibman, M., Khomutov, A., Kizyakov, A., Nitze, I., and Grosse, G.: High-resolution inventory and classification of retrogressive thaw slumps in West Siberia, Earth Syst. Sci. Data Discuss., 2025, 1–30, <https://doi.org/10.5194/essd-2025-164>, 2025.

Nitze, I., Heidler, K., Nesterova, N., Küpper, J., Schütt, E., Hölzer, T., Barth, S., Lara, M. J., Liljedahl, A. K., and Grosse, G.: DARTS: Multi-year database of AI-detected retrogressive thaw slumps in the circum-arctic permafrost region, Scientific Data, 12, 1512, <https://doi.org/10.1038/s41597-025-05810-2>, 2025.

Pekel, J.-F., Cottam, A., Gorelick, N., and Belward, A. S.: High-resolution mapping of global surface water and its long-term changes, Nature, 540, 418–422, <https://doi.org/10.1038/nature20584>, 2016.

Planet Team: Planet imagery product specifications, 2018.

Porter, C., Howat, I., Noh, M.-J., Husby, E., Khuvis, S., Danish, E., Tomko, K., Gardiner, J., Negrete, A., Yadav, B., Klassen, J., Kelleher, C., Cloutier, M., Bakker, J., Enos, J., Arnold, G., Bauer, G., and Morin, P.: ArcticDEM - Mosaics, Version 4.1 (V1), <https://doi.org/10.7910/DVN/3VDC4W>, 2023.

Prentice, R. L. and Gloeckler, L. A.: Regression analysis of grouped survival data with application to breast cancer data, Biometrics, 34, 57–67, 1978.

Riley, S. J., DeGloria, S. D., and Elliot, R.: Index that quantifies topographic heterogeneity, intermountain Journal of sciences, 5, 23–27, 1999.

Sappington, J. M., Longshore, K. M., and Thompson, D. B.: Quantifying Landscape Ruggedness for Animal Habitat Analysis: A Case Study Using Bighorn Sheep in the Mojave Desert, The Journal of Wildlife Management, 71, 1419–1426, <https://doi.org/10.2193/2005-723>, 2007.

Tebaldi, C., Debeire, K., Eyring, V., Fischer, E., Fyfe, J., Friedlingstein, P., Knutti, R., Lowe, J., O’Neill, B., Sanderson, B., van Vuuren, D., Riahi, K., Meinshausen, M., Nicholls, Z., Tokarska, K. B., Hurtt, G., Kriegler, E., Lamarque, J.-F., Meehl, G., Moss, R., Bauer, S. E., Boucher, O., Brovkin, V., Byun, Y.-H., Dix, M., Gualdi, S., Guo, H., John, J. G., Kharin, S., Kim, Y., Koshiro, T., Ma, L., Olivié, D., Panickal, S., Qiao, F., Rong, X., Rosenbloom, N., Schupfner, M., Séférian, R., Sellar, A., Semmler, T., Shi, X., Song, Z., Steger, C., Stouffer, R., Swart, N., Tachiiri, K., Tang, Q., Tatebe, H., Voldoire, A., Volodin, E., Wyser, K., Xin, X., Yang, S., Yu, Y., and Ziehn, T.: Climate model projections from the Scenario Model Intercomparison Project (ScenarioMIP) of CMIP6, Earth System Dynamics, 12, 253–293, <https://doi.org/10.5194/esd-12-253-2021>, 2021.

van der Wiel, K., Beersma, J., van den Brink, H., Krikken, F., Selten, F., Severijns, C., Sterl, A., van Meijgaard, E., Reerink, T., and van Dorland, R.: KNMI’23 Climate Scenarios for the Netherlands: Storyline Scenarios of Regional Climate Change, Earth’s Future, 12, e2023EF003983, <https://doi.org/10.1029/2023EF003983>, 2024.

Wood, S. N.: Generalized Additive Models: An Introduction with R, Second Edition, 2nd ed., Chapman and Hall/CRC, New York, 496 pp., <https://doi.org/10.1201/9781315370279>, 2017.

Zhao, Z., Fan, C., and Liu, L.: Geo SAM: A QGIS plugin using Segment Anything Model (SAM) to accelerate geospatial image segmentation, , <https://doi.org/10.5281/zenodo.8191039>, 2023.
